# NaFSI and NaTFSI Solutions in Ether Solvents from Monoglyme to Poly(ethylene oxide)—A Molecular Dynamics Study

**DOI:** 10.1021/acs.jpcb.1c05793

**Published:** 2021-09-08

**Authors:** Piotr Wróbel, Piotr Kubisiak, Andrzej Eilmes

**Affiliations:** Faculty of Chemistry, Jagiellonian University, Gronostajowa 2, 30-387 Kraków, Poland

## Abstract

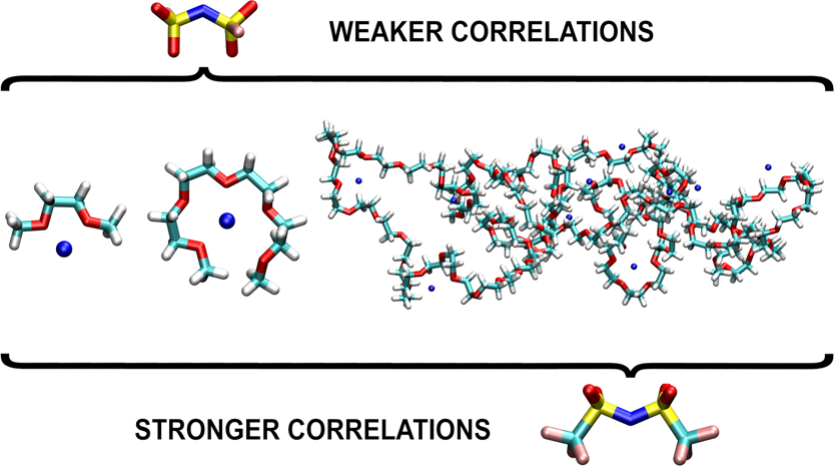

Classical molecular dynamics simulations have been performed for a series of electrolytes based on sodium bis(fluorosulfonyl)imide or sodium bis(trifluoromethylsulfonyl)imide salts and monoglyme, tetraglyme, and poly(ethylene oxide) as solvents. Structural properties have been assessed through the analysis of coordination numbers and binding patterns. Residence times for Na–O interactions have been used to investigate the stability of solvation shells. Diffusion coefficients of ions and electrical conductivity of the electrolytes have been estimated from molecular dynamics trajectories. Contributions to the total conductivity have been analyzed in order to investigate the role of ion–ion correlations. It has been found that the anion–cation interactions are more probable in the systems with NaTFSI salts. Accordingly, the degree of correlations between ion motions is larger in NaTFSI-based electrolytes.

## 1 Introduction

Lithium-ion batteries, commercially available since the 90’s of the 20th century, have dominated the market of portable electronic devices. Nevertheless, new challenges arise for the power-storage technology, caused by the development of electric vehicles and the need for stationary large-scale power-storage utilities, capable of stabilizing the power grids against fluctuating power supply from renewable sources. Issues related to growing demand for lithium salts and to the environmental impact of the technology stimulate interest in “beyond-Li” alternative chemistries, such as sodium-ion devices.^1−5^

Similarly to the Li-based devices, systems investigated as possible electrolytes for Na-ion batteries are sodium salt solutions in molecular solvents, ionic liquids, or mixed solvents.^6−14^ Several of those works are aimed at the study of highly concentrated salt solutions in search for better performing systems.^11−13,15,16^ Among the electrolytes using molecular liquids are the systems based on diglyme^17−19^ or other short glymes as solvents—usually from mono- to pentaglyme.^20−31^ There are also studies on polymer-based systems, offering several advantages, such as better mechanical properties and safety. However, conductivity of solid polymer electrolytes is lower than that of liquid systems. In most cases, poly(ethylene oxide) (PEO) is used as a polymer matrix,^32−34^ although applications of other polymers have also been reported.^35,36^

Vibrational (infrared or Raman) spectroscopy is commonly used to experimentally assess interactions between ions and solvents in Na-conducting electrolytes.^20,21,24,27−29,31,33,34,37^ These findings are supplemented by the results of quantum-chemical calculations.^13,20,21,25,28,38^ Early works performing molecular dynamics (MD) for polymer electrolytes with Na^+^ ions were focused on glyme–NaI^39,40^ or PEO–NaI^41,42^ systems. Nowadays, classical and ab initio MD simulations are routinely applied to support and elucidate measurements of transport properties and structure of electrolytes.^15,24,29,31,36,37,43−45^

Sodium bis(fluorosulfonyl)imide (NaFSI) or sodium bis(trifluoromethylsulfonyl)imide (NaTFSI) were used as sodium salts in many of the experimentally investigated electrolytes based on ethers or PEO.^20,21,25,26,28,32−34^ Nevertheless, MD simulations focused usually on only one of these anions and either on short oligoglyme molecules or on polymer chains. The applied methodology differs between these works, making direct comparison of results rather difficult. Therefore, in this study, we attempted to systematically compare results of simulations obtained within the same classical MD force field (FF), for both anions and for different lengths of the ether chain.

To this end, we used classical, polarizable FF for systems with monoglyme, tetraglyme, and short PEO chains as solvents, loaded with NaFSI or NaTFSI at the same Na/O ratios. For this series of simulations, we will compare the structure of electrolytes [coordination numbers (CNs) and binding patterns] and their dynamics (ion residence times and transport properties) to assess the effects of changes in the salt anion and in the length of solvent molecules. The results will be discussed with respect to available experimental data.

## 2 Computational Methods

Electrolytes investigated in this work were the NaFSI or NaTFSI solutions in monoglyme, tetraglyme, or short PEO chains containing 100 oxygen atoms (molecular weight Mw = 4.4 × 10^3^ g/mol). The total number of ether oxygen atoms in each system was equal to 600. Accordingly, the numbers of solvent molecules were 300, 120, and 6 for monoglyme, tetraglyme, and PEO, respectively. Two salt concentrations were used, with 30 or 100 ion pairs in the simulation box, yielding the Na/O ratios 1:20 or 1:6. These two ratios were examined in experiments on PEO/Na(T)FSI electrolytes.^33^ Initial structures of the electrolytes with small solvent molecules were prepared using Packmol^46^ program, whereas for amorphous PEO systems, polymer builder and amorphous builder from the Scigress^47^ package were used. For each system composition, we performed 10 independent simulations.

Classical polarizable FF was used in MD simulations with polarization effects modeled via Drude particles^48^ attached to all nonhydrogen atoms in the system, excluding Na^+^ ions. Van der Waals potential parameters and bonded parameters for glyme molecules and PEO were taken from the study on FF for PEO simulations,^49^ except parameters for dihedral angles, which were transferred from the GAFF parameterization^50,51^ in the case of monoglyme or from the Amber FF for organic compounds^52^ in the case of tetraglyme and PEO. For all solvent molecules, polarizabilities and partial charges of atoms were taken from the work on polarizable simulations of PEO.^53^ It should be noted that the nonbonded parameters, most important for cation–solvent interactions, are the same for all solvents.

Bonded parameters for TFSI and FSI anions were taken from the Canongia Lopes/Pádua FF,^54,55^ and the polarizabilities and partial charges were based on APPLE&P parameterization.^56^ Van der Waals parameters for TFSI and FSI were taken from the Köddermann’s work^57^ and APPLE&P FF, respectively. Parameters for Na^+^ were adapted from ref (58). Additionally, we modified the van der Waals parameters for the pair of Na^+^ cation and O atom from TFSI in order to improve the geometry of the Na^+^–TFSI complexes with respect to quantum-chemical results. All parameters of the FF used in this work are listed in Supporting Information.

MD simulations were performed in NAMD v 2.12 simulation package.^59^ Investigated systems were equilibrated for approximately 50 ns (glymes) or 100 ns (PEO) in the NpT ensemble at *p* = 1 atm and *T* = 303 K with Langevin dynamics and modified Nose–Hoover Langevin barostat.^60,61^ Then, 150–250 ns of trajectories were obtained in the NVT ensemble at the density obtained at the NpT stage; shorter simulation times (150–200 ns) were used for monoglyme and tetraglyme and longer (reaching 250 ns) for PEO systems. A time step of 1.0 fs was used to integrate the equations of motion. Periodic boundary conditions were used, and electrostatic interactions were taken into account via the particle mesh Ewald algorithm.^62^ Results for a given kind of the system were averaged over 10 independent MD trajectories.

## 3 Results

### 3.1 Structure of Electrolytes

**Figure 1:**
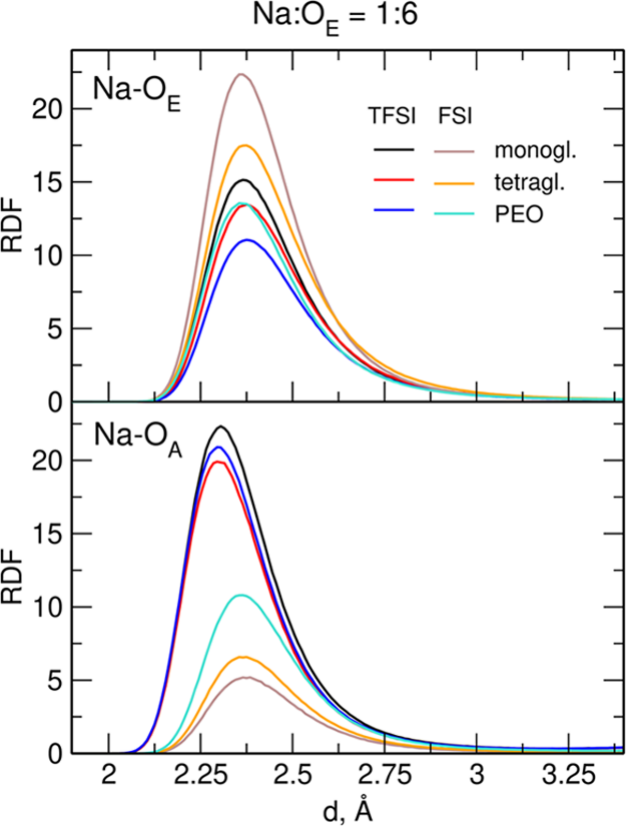
RDFs for Na–O atom pairs in the 1:6 electrolytes.

In Figure 1, we show the radial distribution functions (RDFs) for Na–O_E_ and Na–O_A_ atom pairs (where O_E_ and O_A_ denote the oxygen atom from the solvent molecule or the anion, respectively), calculated for systems with a Na/O_E_ ratio equal to 1:6. The plot for the ratio 1:20 is available in Supporting Information (Figure S1). The position of the Na–O_E_ RDF maximum does not depend much on the anion, solvent molecule, or salt concentration, and in all systems, it is located at 2.36–2.37 Å. Its height in the electrolytes based on NaFSI is larger than that for the systems with NaTFSI. Conversely, the distance at which the maximum of Na–O_A_ RDF appears depends on the anion. For FSI anions, the position of the maximum is 2.36–2.37 Å, that is, at the same distance as for cation–ether oxygen. Distances to TFSI anions are shorter, and the Na–O_TFSI_ maximum is located at 2.3 Å. Na–O_FSI_ RDF values at maximum are significantly smaller than those obtained for Na–O_TFSI_, especially for the systems with mono- or tetraglyme at low salt concentrations. The difference in the height of RDF maxima suggests that Na^+^ complexation by the FSI anions is weaker, especially in the solutions with short glymes as a solvent.

**Figure 2:**
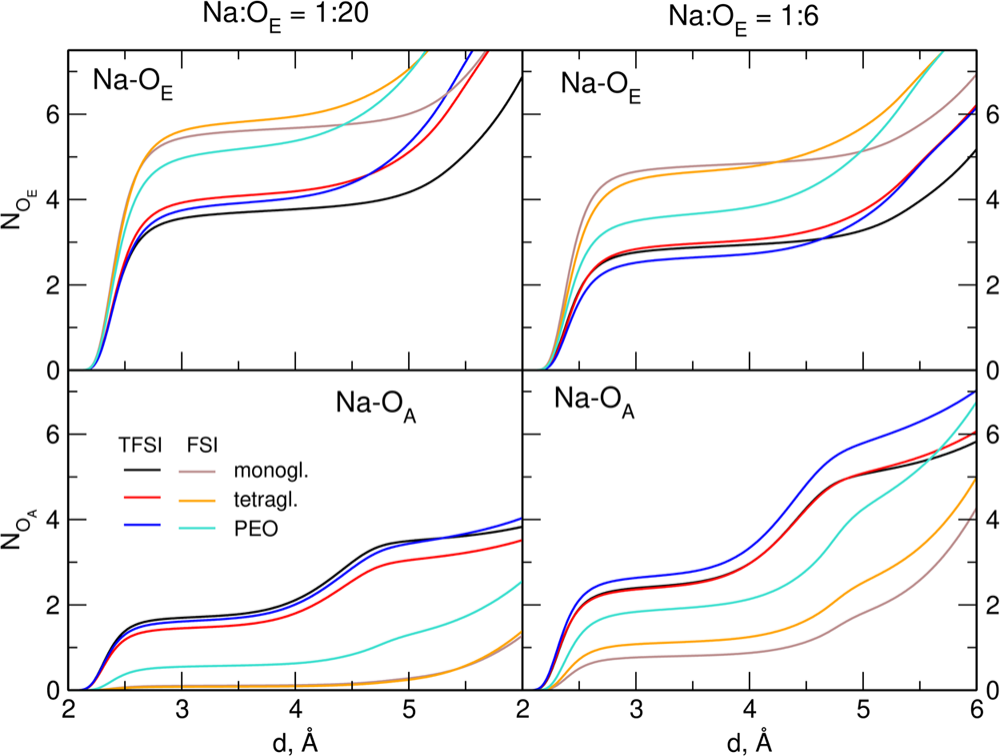
Integrated RDFs for 1:20 and 1:6 electrolytes.

The latter effect is readily seen from the integrated RDFs (running CNs) shown in Figure 2. Average numbers of O_E_ and O_A_ atoms found at the 3.5 Å distance from the Na^+^ ion are collected in Table 1. At low salt load in the systems with small solvent molecules, coordination of the cation to FSI anions is marginal. It increases in the NaFSI 1:20 PEO-based electrolyte; however, even in this system, there is, on average, less than one O_A_ atom coordinated to the Na^+^ ion. Accordingly, CNs for ether oxygen atoms are the largest in 1:20 systems with NaFSI. With the salt concentration increased to the Na/O_E_ ratio equal 1:6, Na–O_A_ CNs increase at the expense of cation–solvent coordination. Nevertheless, it is clear that the cation coordination to the ether molecule is preferred over interaction with anions. Only in the NaTFSI–PEO 1:6 electrolyte, the number of ether oxygen atoms in the solvation shell of the cation is slightly smaller than the number of oxygen atoms from TFSI anions. The last column of Table 1 shows that the total number of coordinating oxygen atoms (regardless of their provenance) is fairly constant and varies between 5.4 and 5.9. Lower values are observed in the systems with substantial Na-anion coordination, whereas the highest total CN of Na^+^ is reached in the NaFSI-based electrolytes with negligible cation–anion coordination. From these data, we can conclude that the use of NaFSI salt promotes cation–solvent interactions. On the other hand, a higher degree of cation–anion coordination is found in the electrolytes with PEO as the solvent, with an exception of the NaTFSI–PEO 1:20 electrolyte, for which the average number of O_A_ atoms is slightly smaller than in the corresponding monoglyme solution (1.70 and 1.78, respectively).

**Table 1:** Average Numbers of O_E_ and O_A_ Atoms and the Total Number of O Atoms within the 3.5 Å Distance from the Na^+^ Ion

system	*N*_OE_	*N*_OA_	*N*_tot_
NaTFSI–monoglyme 1:20	3.69	1.78	5.47
NaTFSI–monoglyme 1:6	2.87	2.52	5.39
NaFSI–monoglyme 1:20	5.60	0.11	5.71
NaFSI–monoglyme 1:6	4.78	0.81	5.59
NaTFSI–tetraglyme 1:20	4.09	1.53	5.62
NaTFSI–tetraglyme 1:6	2.97	2.50	5.47
NaFSI–tetraglyme 1:20	5.81	0.08	5.89
NaFSI–tetraglyme 1:6	4.64	1.14	5.78
NaTFSI–PEO 1:20	3.91	1.70	5.61
NaTFSI–PEO 1:6	2.64	2.79	5.43
NaFSI–PEO 1:20	5.18	0.58	5.76
NaFSI–PEO 1:6	3.66	1.94	5.60

**Figure 3:**
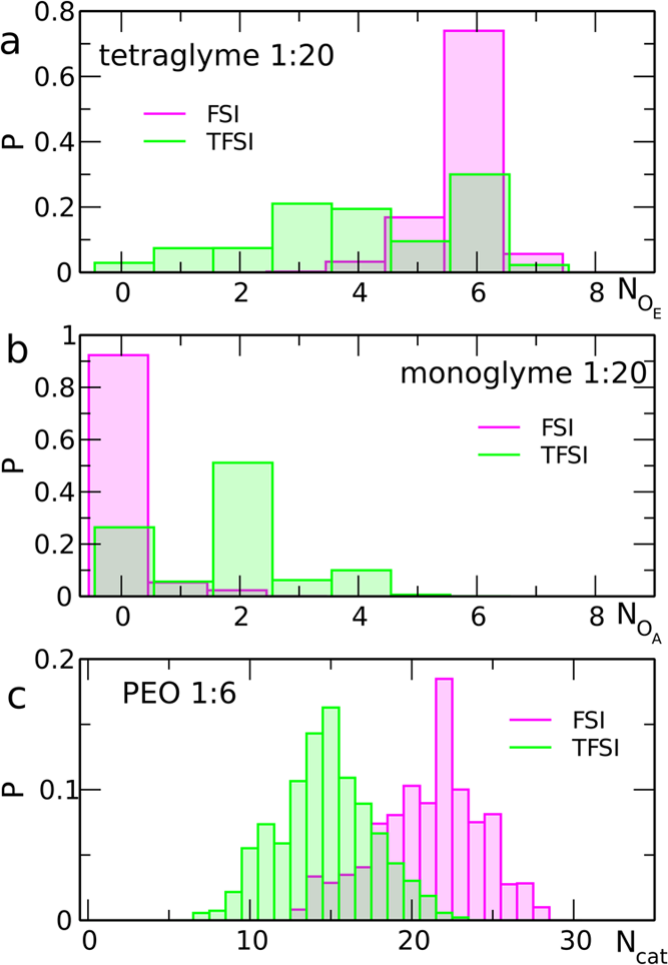
Distributions of the number of ether oxygen atoms coordinated to Na^+^ (a), of the number of anion oxygen atoms coordinated to Na^+^ (b), and of the number of Na^+^ ions coordinated to PEO molecules (c).

In addition to the average CNs, some more insights into the structure of cation–solvent or cation–anion aggregates can be obtained from the distributions of CNs and the distributions of the solvent numbers (SNs), that is, the number of solvent molecules coordinating the cation. Histograms of several types of CNs and SNs for all investigated electrolytes are provided in Supporting Information (Figures S2–S8), and selected representative examples are shown in Figure 3.

The most probable total number of oxygen atoms coordinated to the Na^+^ ion is 5 or 6 (Figure S2). The higher value is preferred at low salt load and in the systems with FSI anions, whereas in the NaTFSI-based electrolytes, abundance of both values is similar and the CN = 5 becomes more probable in the 1:6 systems. The SNs (Figure S3) decrease in the order monoglyme > tetraglyme > PEO, as a consequence of increasing number of oxygen atoms available for coordination in the ether molecule. One may also note that the most-probable SN in NaFSI electrolytes is higher than that in the NaTFSI systems, reaching the value of 3 in the former and the value of 2 in the latter. Therefore, in the 1:20 NaFSI–PEO electrolyte, most cations interact with two PEO chains, whereas at the same concentration in the NaTFSI system, coordination to a single chain is more probable. The difference between FSI and TFSI-based systems may be attributed to increased probability of cation–anion interaction in the latter, reducing the number of ether oxygen atoms coordinating the cation.

Histograms of the number of O_E_ atoms interacting with Na^+^ (Figure S4) readily differ between systems with different anions; an example for 1:20 electrolyte with a tetraglyme solvent is shown in Figure 3a. In the 1:20 NaFSI electrolyte, the CN = 6 is the most probable (70% in mono- or tetraglyme and 45% in PEO). At the 1:6 concentration, the distribution of CNs becomes wider. In monoglyme, two CN values, six and four, are still dominant, but in tetraglyme, abundance of a given CN decreases from CN = 6 to CN = 1. In PEO, all CN values between two and six have a similar probability. There are no such significant changes in NaTFSI systems. The distributions are always broad, and the abundance of CNs = 3, 4, or 6 is comparable, both at 1:20 and 1:6 salt load; in the more concentrated electrolyte, the probability shifts toward lower CN values.

The results described above are correlated to the distributions of the number of anions (Figure S5) and the number of O_A_ atoms (Figure S6) coordinating the Na^+^ ion. A sample histogram comparing monoglyme 1:20 systems with different anions is shown in Figure 3b. In diluted NaFSI electrolytes, the majority of cations do not interact directly with FSI anions. At higher salt concentration, the maximum of the probability distribution shifts toward the value corresponding to one anion coordinated to the cation. Accordingly, in 1:6 NaFSI systems with monoglyme, the most-probable value of the CN for O_A_ atoms equals zero and increases to one for tetraglyme and PEO. In the latter case, all O_A_ values between 0 and 2 are approximately equally probable. In the solutions of NaTFSI, interactions of a cation with one or two anions are most frequent, although the cases of lack of anion coordination are still quite probable. Therefore, the distribution of O_A_ CNs is broad and, depending on the system, maximum values reached are 4 or 5. It can be seen in Figure S6 that even values (0, 2, or 4) of O_A_ are preferred in NaTFSI electrolytes, indicating that the coordination of Na^+^ to TFSI anion is bidentate.

Finally, we also calculated the histograms showing the number of different Na^+^ ions interacting with one solvent molecule (Figure S7, a sample for the PEO 1:6 electrolyte is shown in Figure 3c) or the number of oxygen atoms from the solvent molecule, which are engaged in the coordination of the cation (Figure S8). In 1:20 solutions with monoglyme or tetraglyme used as the solvent, about 50–80% of solvent molecules are free, that is, do not interact with cations. In concentrated electrolytes, however, most solvent molecules coordinate one or two Na^+^ ions; higher values are more probable in NaFSI solutions. Accordingly, in 1:6 NaFSI solutions in mono- and tetraglyme, a large number of solvent molecules use all their oxygen atoms (2 or 5, respectively) for coordination. The corresponding values are smaller in NaTFSI electrolytes; for example, in the 1:6 NaTFSI solution in tetraglyme, the most probable number of O_E_ atoms used by a glyme molecule for cation coordination equals three.

In the PEO electrolytes, there are no major differences between TFSI and FSI-based systems in the number of cations coordinated to a single polymer chain, although a small shift of the histograms to higher CNs is noticeable for 1:20 PEO–NaFSI. In the concentrated 1:6 electrolyte, the difference is better pronounced. The distribution of the number of complexed cations has the maximum at about *N*_cat_ = 15 in the case of PEO–NaTFSI, whereas for PEO–NaFSI, it appears well above *N*_cat_ = 20. Accordingly, the maxima in the histograms of the number of coordinating O_E_ atoms are at about *N*_at_ = 40 or 60 for NaTFSI and NaFSI electrolytes, respectively. Such a difference is related to the increased probability of Na^+^ coordination to the anion in the electrolytes with TFSI salt, resulting in less cation interactions with the ether molecule. It may be noted that at the 1:6 salt load, about half of the oxygen atoms from PEO molecules is involved in the interactions with sodium cations.

The total number of coordinating oxygen atoms (*N*_tot_ in Table 1) decreases with increasing salt concentration. Such an effect was observed for total CNs deduced from Raman spectra of NaTFSI solutions in carbonate solvents^63^ or mixed molecular/ionic liquid electrolytes.^64^ Steric effects hindering both solvents and TFSI to simultaneously coordinate the Na^+^ ion were the suggested origin of these observations.^64^

CNs for monoglyme-based electrolytes can be compared to the results of other simulations. In ref (30), polarizable MD simulations were used for NaTFSI solutions and the trend of increasing cation coordination to anions was observed for increasing salt concentration. The same trend is visible from our data in Table 1; however, our results predict a little larger CNs for Na–O_A_ pairs: 1.8 and 2.5 for 1:20 and 1:6 electrolytes, respectively, whereas the results of ref (30) for the corresponding molar concentrations are about 1.2 and 1.8. Accordingly, our CNs for O_E_ atoms are smaller than those found in ref (30). For NaFSI–monoglyme, we also observe an increase in cation–anion coordination at higher salt load, but the changes are slower than those calculated from the density-functional tight-binding MD,^43^ and our values are close to those obtained for dilute systems in ref (43).

The preference of the sodium ion to coordinate to ether oxygen atoms rather to the anions observed in our simulations is in agreement with the results of an MD study on a TFSI-derived ionomer with ether spacer groups: with increasing length of spacers, Na^+^ coordination to ether O atoms increased and the coordination to TFSI oxygen atoms decreased.^65^

There is a discrepancy between our simulations and the conclusions about (T)FSI coordination in PEO drawn from the Raman spectra in ref (33). According to those results, at low salt load, almost all TFSI anions are “free”, whereas only about 2/3 of FSI do not interact with Na^+^. At higher salt concentrations in 1:6 systems, the amount of “free” anions becomes similar but still with a noticeable preference of FSI to form aggregates with cations. CNs obtained in our simulations listed in Table 1 suggest the opposite: the TFSI anion is more likely to form an ion pair. In Figure S9, we compared the histograms of the number of Na^+^ ions coordinated to anions, and indeed, the number of free anions in 1:20 systems for FSI solutions is larger than that for TFSI, in accordance with CNs in Table 1, but in disagreement with ref (33). However, other MD simulations for Na(T)FSI solutions in monoglyme^30,43^ show that (1) at low salt concentrations, TFSI anions also interact with cations and (2) probability of Na–FSI interactions decreases substantially with ion concentration. Likewise, it was concluded from Raman spectra of NaFSI solutions in tetra- and pentaglyme that in dilute solutions, FSI anions are not involved in the coordination to cations.^20^ It is therefore unclear why these trends may change in PEO electrolytes, in which TFSI anions are reported to be almost completely uncoordinated to anions at low concentration. From the point of view of MD simulations, there is a possibility that the FF parameterization for PEO should differ from that for glyme molecules or that the simulation time was still too short to complete the structural change in the initially amorphous sample.

**Figure 4:**
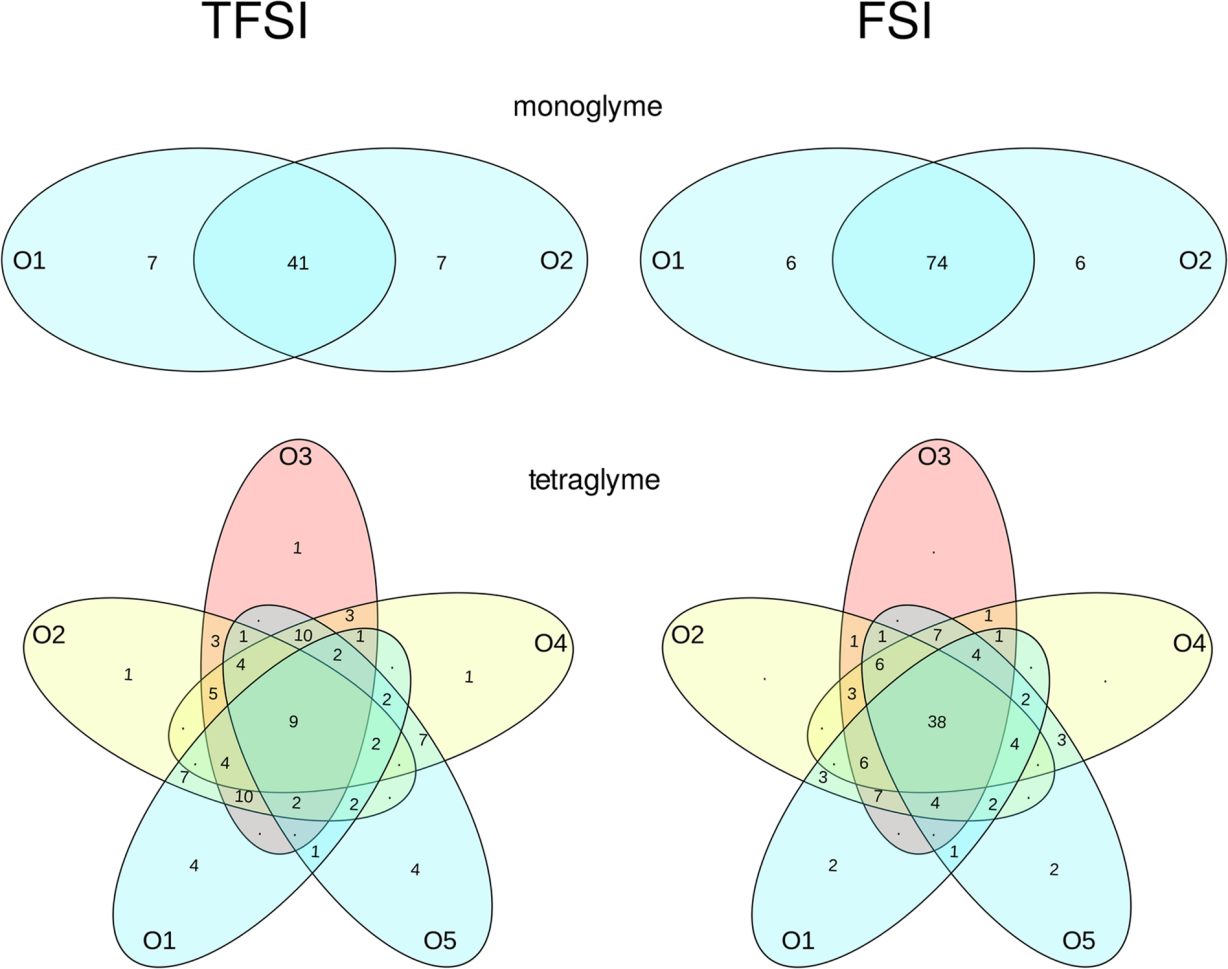
Venn diagrams showing the connectivity between the Na^+^ ion and the O atoms of the monoglyme and tetraglyme molecules in 1:6 electrolytes. Values lower than 1% are displayed as dots.

In order to analyze in more detail, the cation-binding pattern of mono- and tetraglyme, we produced Venn diagrams (Figure 4 for 1:6 systems and Figure S10 for 1:20 electrolytes), showing the percentage of solvent molecules engaging specified subset of O_E_ atoms in interactions with Na^+^ cations (one cation or more). The graphs were symmetrized with respect to equivalent atoms. For example, values shown for monoglyme–NaTFSI in Figure 4 mean that 41% of solvent molecules interact with cations via both oxygen atoms, whereas 14% uses only one O_E_ atom for coordination; the latter value was equally divided between two indistinguishable atoms O_1_ and O_2_.

In the 1:20 monoglyme-based electrolytes, the probability of solvent–cation interactions through both O_E_ atoms of glyme molecule increases from 16 to 26% between NaTFSI and NaFSI systems. The differences between TFSI and FSI diagrams for tetraglyme at this salt concentration are small, with only a slightly increased probability of cation coordination in NaFSI solution.

Much larger differences can be seen for 1:6 electrolytes in Figure 4. In the monoglyme solutions, the percentage of ether molecules interacting through both oxygen atoms almost doubles in the NaFSI electrolyte (41 and 74% for NaTFSI and NaFSI systems, respectively). Likewise, the number of tetraglyme molecules interacting with Na^+^ using all five O_E_ atoms in the NaFSI solution is four times larger than in the NaTFSI electrolyte (38 and 9%, respectively). In the tetraglyme–NaTFSI system, more probable (20%) is the configuration in which the tetraglyme molecule coordinates the cation(s) via three consecutive atoms O1, O2, and O3 (or equivalent). In NaFSI electrolytes, it is less abundant (14%). If the tetraglyme molecule interacts with cations via more than one O_E_ atom, in more than 80% of cases, the interacting atoms are the consecutive oxygen atoms from the molecule. It may be noted that the amount of tetraglyme molecules having all oxygen atoms connected to Na^+^ ions is much smaller than the 82% presented in Venn graphs for 1:5 LiTFSI solution in tetraglyme.^66^ This difference can be rationalized by stronger glyme interactions with smaller Li^+^ cations.

For practical reasons, it is not possible to visualize the binding pattern of PEO using Venn diagrams. To get some insights into this issue, we plotted in Figure S11 the probability that the same Na^+^ cation, which interacts with the oxygen atom at the site *N* = 0, is coordinated to an oxygen atom positioned Δ*N* sites apart. Apparently, the most probable is Na^+^ coordination to few neighboring O atoms: the probability is substantial only for atoms being not farther away than three sites. For more distant atoms, the probability is less than 1%. Nevertheless, there are also several Δ*N* values larger than 20 (usually with the probability less than 0.1%). They correspond to the cases where a loop of the PEO chain enables coordination of Na^+^ ions simultaneously to different parts of the chain.

### 3.2 Dynamics

In order to assess the time scale of the dynamics of cation–solvent interactions, we used the MD trajectories to calculate the residence time autocorrelation function
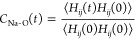
1where *H*_*ij*_(*t*) = 1 if at time *t*, the distance between the *i*th Na^+^ ion and the *j*th O atom is smaller than a threshold value or *H*_*ij*_(*t*) = 0 otherwise. Threshold values were 3.5 Å for O_E_ and 3.0 Å for O_A_ atoms. The autocorrelation functions were obtained separately for ether O_E_ and anion O_A_ oxygen atoms. In addition, the residence functions of solvent molecules *C*_Na-E_(*t*) or of anions *C*_Na-an_(*t*) were calculated assuming *H*_*ij*_(*t*) = 1 if at time *t*, any of the oxygen atoms from the *j*th ether molecule/anion is coordinated to the *i*th cation. For a more quantitative description, the stretched exponential functions exp [−(*t*/τ_O_)^α^] were fitted to *C*_Na–O_(*t*), yielding the oxygen atom residence times τ_O_. Likewise, the solvent and anion residence times τ_E_ and τ_an_ were obtained from the fits to *C*_Na-E_(*t*) and *C*_Na-an_(*t*), respectively.

**Figure 5:**
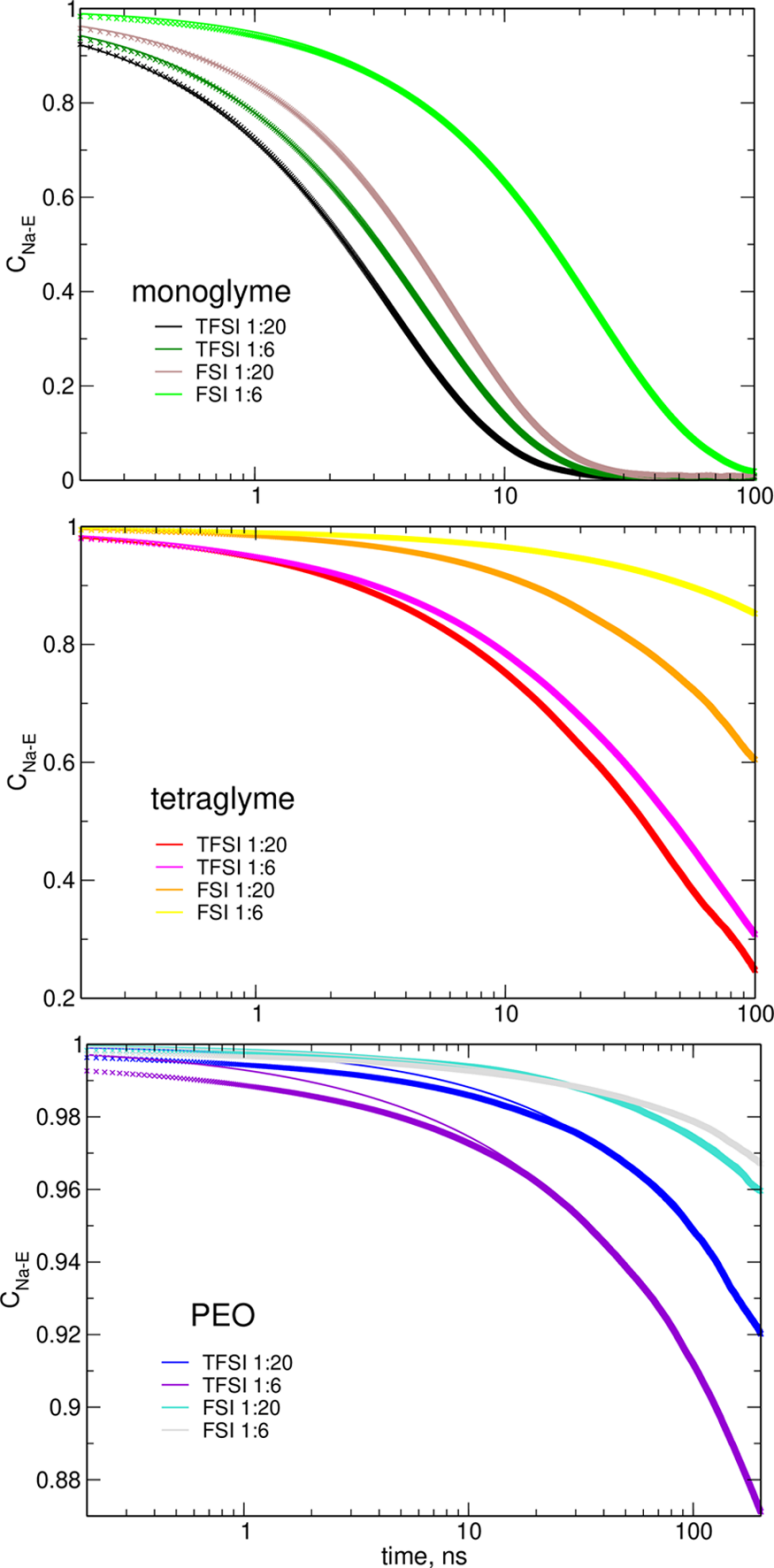
Na-ether molecule residence time autocorrelation function. Lines are fits to the data.

The Na^+^ solvent molecule autocorrelation functions for studied systems are shown in Figure 5. Plots for cation–anion and cation–oxygen atom functions are available in Supporting Information (Figures S12–S14). Estimated residence times are listed in Table 2. Dynamics of the system becomes significantly slower in more viscous electrolytes based on tetraglyme and PEO, and only in monoglyme solutions, autocorrelation functions reached the zero value within the time of the MD simulations. In order to assess how it affects the estimated residence times, we performed a test using the monoglyme data. We truncated the data at the values of the autocorrelation function corresponding to those reached for tetraglyme and PEO and then fitted the residence times and compared to the results computed for full data sets. The values of τ obtained from the truncated data were always larger than the original estimates. For tetraglyme, the difference reached 5%; the overestimate for PEO systems amounted to 15 or 20–25% for the solvent or oxygen atoms residence times, respectively. Uncertainty of estimated residence times increases therefore in the order monoglyme < tetraglyme < PEO, and the values obtained for PEO are systematically overestimated.

**Table 2:** Residence Times, Diffusion Coefficients, and Conductivity of Electrolytes

system	τ_OA_, ns	τ_an_, ns	τ_OE_, ns	τ_E_, ns	*D*_+_, m^2^/s	*D*_–_, m^2^/s	σ, S/m
NaTFSI–monoglyme 1:20	3.7	18.4	3.1	3.5	1.2 × 10^–10^	1.2 × 10^–10^	0.22
NaTFSI–monoglyme 1:6	3.9	13.3	4.2	4.7	4.9 × 10^–11^	4.5 × 10^–11^	0.17
NaFSI–monoglyme 1:20	0.19	0.44	5.7	6.1	9.6 × 10^–11^	1.2 × 10^–10^	0.61
NaFSI–monoglyme 1:6	0.78	3.1	21	23	1.1 × 10^–11^	1.2 × 10^–11^	0.20
NaTFSI–tetraglyme 1:20	14	85	39	61	4.3 × 10^–12^	5.3 × 10^–12^	1.7 × 10^–2^
NaTFSI–tetraglyme 1:6	22	92	48	79	2.2 × 10^–12^	2.4 × 10^–12^	1.2 × 10^–2^
NaFSI–tetraglyme 1:20	1.2	8.0	140	250	1.3 × 10^–12^	2.1 × 10^–12^	1.1 × 10^–2^
NaFSI–tetraglyme 1:6	16	190	1560	1710	1.6 × 10^–13^	1.9 × 10^–13^	2.3 × 10^–3^
NaTFSI–PEO 1:20	850	7000	3100	9090	4.7 × 10^–14^	4.7 × 10^–14^	2.3 × 10^–4^
NaTFSI–PEO 1:6	760	2920	2400	7020	7.3 × 10^–14^	7.0 × 10^–14^	3.9 × 10^–4^
NaFSI–PEO 1:20	94	3050	6790	27500	2.9 × 10^–14^	2.9 × 10^–14^	2.5 × 10^–4^
NaFSI–PEO 1:6	250	16100	32000	98100	1.8 × 10^–14^	1.6 × 10^–14^	3.7 × 10^–4^

We can draw some general conclusions from the data in Table 2 and Figures 5, S12–S14. Of course, residence times increase significantly with increasing length of the ether molecule. Residence times calculated for monoglyme solutions can be compared to other MD results. The O_E_ and O_A_ residence times in monoglyme/NaTFSI electrolytes are in the range of 3–4 ns. Values of residence times for Na^+^–O bonds found in ref (30) are somewhat smaller (1–2 ns). The O_A_ residence times of about 0.2 ns calculated for the NaFSI solution in monoglyme are similar to the 120 ps lifetime of the solution structure estimated in ref (43); however, in the latter work, there was no increase in the lifetime observed for the concentrated electrolyte, whereas such an effect can be seen in our data in Table 2.

The calculated O_A_ residence times for NaFSI electrolytes are always smaller than those for the corresponding NaTFSI systems. It is, however, not true when the residence time of the anion as a whole is considered: in the 1:6 NaFSI solutions in tetraglyme and PEO, values of τ_an_ are larger than those estimated for TFSI-based counterparts. For all systems, residence times of ether oxygen atoms and residence times of solvent molecules in NaFSI electrolytes are larger than in NaTFSI solutions. This difference is the largest for the solvent residence times in the concentrated tetraglyme electrolytes: τ_E_ of 1:6 NaFSI solution is more than 20 times larger than τ_E_ of the 1:6 NaTFSI system. Therefore, the FSI salt seems to promote a more stable Na^+^ binding to the solvent and a slower exchange of ions in the solvation shell.

An interesting effect is observed for PEO-based electrolytes. In NaFSI solutions, all residence times increase significantly in the 1:6 electrolyte compared to the 1:20 solution. For NaTFSI systems, the change is opposite: residence times are larger in less-concentrated solutions, suggesting that the mechanism of ion transport may be different, depending on the anion of Na salt. A possible explanation can be related to a larger number of cation–anion interactions in NaTFSI systems which may increase the probability of Na^+^ exchange between anions, providing an alternative way of cation motion in addition to hopping between PEO oxygen atoms. Two mechanisms of charge transport, diffusion of free ions and hopping between ion pairs, were postulated for concentrated Na electrolytes in ether solvents.^29^ A similar effect of residence times decreasing with increasing salt concentration was also reported for sodium salt solutions in acetonitrile^45^ and attributed to the transport mechanism.

Diffusion coefficients were calculated from the slope of the time dependence of mean square displacement of ion *i*
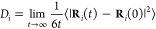
2

Conductivity of the electrolyte was calculated using the Einstein formula as
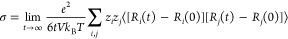
3

In the abovementioned formulas, *t* stands for the time, *V* is the volume of the simulation box, *k*_B_ is the Boltzmann’s constant, *T* is the temperature, *e* is the elementary charge, *z*_*i*_ and *z*_*j*_ are the charges of ions *i* and *j*, R_*i*_(*t*) is the position of *i*th ion at time *t*, and the brackets ⟨⟩ denote the ensemble average.

A way to estimate *D* or σ values using eqs 2 or 3 is to plot the mean displacements and then to find the slope of the linear part of the plot. At short times, ion transport in viscous systems is subdiffusive; therefore, the long-time part of the plot should be used to analyze the diffusive behavior. However, averaging of the data for long times is worse because of less-possible choices of time intervals Δ*t* within the length of the trajectory. The choice of the time window has to be a compromise between the quality of averaging and the possibility to approach the diffusive regime. In our estimates, we used the time ranges of 20–40 ns and 60–100 ns for electrolytes based on short glymes and PEO, respectively.

Estimated values of diffusion coefficients of ions and of the electrical conductivity are collected in Table 2. Changes in diffusion coefficients of ions follow a similar pattern to that observed for solvent residence times—an increase in τ_E_ is accompanied by a decrease in *D*_±_. The most pronounced is the decrease in ion mobility with the increasing size of the solvent molecule, as a result of increasing viscosity of the electrolyte. Diffusion coefficients in NaFSI electrolytes are always smaller than the corresponding values in NaTFSI-based systems. It can be also noted that in most cases, an increase in the salt load from 1:20 to 1:6 results in a decrease in diffusion coefficients. This effect is larger for monoglyme and tetraglyme solutions where the change estimated for NaFSI electrolytes may reach an order of magnitude; for PEO–NaFSI systems, the decrease is smaller. The PEO–NaTFSI electrolytes are an exception—mobility of ions is larger in the more-concentrated electrolyte. This behavior corresponds to the differences in residence times discussed earlier.

**Figure 6:**
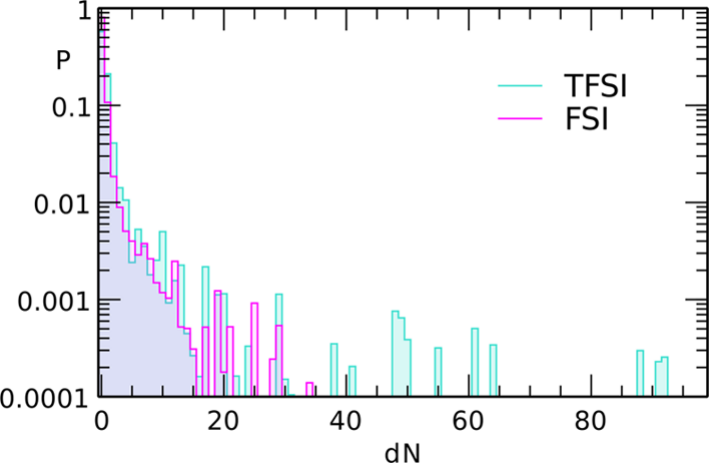
Probability of the displacement of Na^+^ ions by d*N* sites along the PEO chain in 1:6 Na(T)FSI/PEO electrolytes after 250 ns of simulations.

In the case of the PEO-based electrolytes, not only the spatial displacements of sodium cations (given by the diffusion coefficients) are of interest but also the information on how fast the ions move along the polymer chain. In Figure 6, we display for 1:6 Na(T)FSI/PEO systems, the probability that the Na^+^ ion has moved from its original position by d*N* repeat units after 250 ns of simulations. Displacements of the vast majority of ions are small and do not exceed five binding sites. Probabilities of displacements in the range of 5–15 units are below 1%. We may attribute such changes in binding sites to the motions of ions along the chain. There are also less probable cases of quite large d*N* values (up to tens of sites), corresponding to the cases of breaking the ion–PEO interaction at one site and association of the ion to another binding atom in the proximity due to the spatial loop of the PEO chain. These large d*N* changes are more probable in the electrolyte with NaTFSI.

Conductivity of the electrolyte depends mainly on the solvent and decreases in the order monoglyme > tetraglyme > PEO, accordingly to increasing viscosity of the system. Looking at individual solvents, we may note that in short glymes, an increase in salt concentration from 1:20 to 1:6 ratio decreases the conductivity, whereas the change is opposite for PEO. The effect of anion depends on the solvent and salt concentration. Experimental values of the conductivity of monoglyme solutions in the range of concentrations studied here are larger than our results: 1.3–1 S/m for NaTFSI^30^ and 1.84–1.32 S/m for NaFSI.^26^ Values obtained in the experiment^33^ for 1:20 and 1:6 PEO systems at the temperature used in our MD simulations are about 2 × 10^–4^ S/m, except the 1:6 NaFSI electrolyte for which a smaller conductivity of 2 × 10^–5^ S/m was measured. The latter effect was attributed to glass transition of the system. Therefore, our MD estimates are close to the experimental data on PEO conductivity.

Looking at the data in Table 2, one can note an interesting behavior of monoglyme solutions: at a given concentration, the change in the anion from TFSI to FSI decreases ion mobilities but increases the conductivity of the electrolyte. If there were no correlations between ion motions, the conductivity would be proportional to the average of D_+_ and D_–_. Therefore, the observed effect suggests the importance of correlations in our systems.

In order to analyze the correlations in more detail, we partitioned the conductivity given by eq 3 into contributions according to indices *i* and *j* in the sum
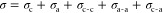
4

The diagonal (*i* = *j*) terms σ_c_ and σ_a_ are related to self-diffusion of cations and anions, respectively. The other three terms are off-diagonal (*i* ≠ *j*) and arise from correlations between different ions: cation–cation (σ_c–c_), anion–anion (σ_a–a_), and cation–anion (σ_c–a_). In the literature, the diagonal terms σ_c_ and σ_a_ are often named “self”-contributions, and the off-diagonal terms related to ions of the same charge σ_c–c_ and σ_a–a_, are referred to as “distinct” cation–cation and anion–anion contributions.

**Figure 7:**
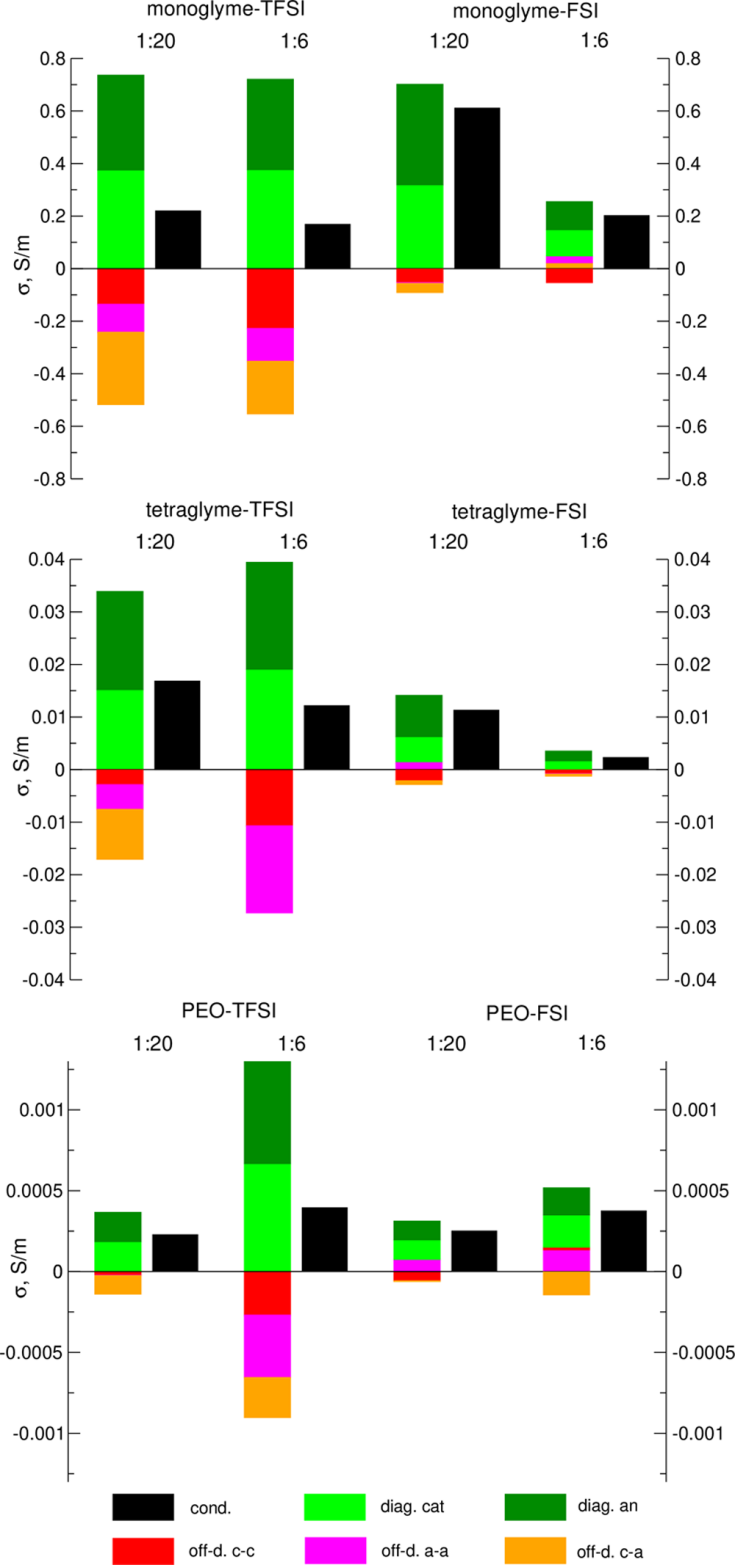
Different contributions (color bars) to the total conductivity (black bars) of electrolytes.

Different contributions to the conductivity of simulated systems are shown in Figure 7, along with their sum giving the total conductivity. The diagonal cation and anion contributions, being sum of squares, are always positive. Their size depends on the diffusion coefficients and the number of ions. Therefore, in PEO and in NaTFSI solutions in shorter glymes, smaller diffusion coefficients in 1:6 electrolytes are compensated by the increase in the concentration of charge carriers and the diagonal contribution to σ increases. However, in the case of NaFSI solutions in mono and tetraglyme, the decrease in D_±_ is larger than the change in concentration and the diagonal contributions to the 1:6 systems decrease.

For a salt solution in a molecular liquid, the off-diagonal terms are negative: motions of ions of the same charge are anticorrelated (assuming that there is no significant amount of multiion aggregates, observed, e.g., in NaI electrolytes^40,67^), whereas cation–anion motions are positively correlated. In all cases, the product of ion charges and displacements in eq 3 is negative; therefore, the ion–ion correlations are expected to contribute toward the decrease in the conductivity. It can be seen in Figure 7 that in general, this is the case in our systems: if the off-diagonal contributions are large, they are negative. Positive values are either close to zero or prone to large errors (in PEO systems).

We can see the difference in the role of correlations in mono- and tetraglyme, depending on the salt anion. Correlations are quite important in NaTFSI solutions where they cancel a large part of the diagonal contributions of ions. A similar effect was observed in MD simulations of NaTFSI in monoglyme,^30^ where the ratio of the ionic conductivity to the uncorrelated ionic conductivity was about 0.5. Conversely, in NaFSI electrolytes, the effect of correlations is small and the conductivity results mainly from “self”-contributions. This explains the difference between monoglyme electrolytes: diffusion coefficients in NaTFSI solution are similar or larger than those in NaFSI electrolytes, but large correlations in the former system reduce the conductivity below the value estimated for NaFSI systems. The role of correlations decreases in the 1:20 NaTFSI/PEO system, but they are still important in the 1:6 electrolyte.

### 3.3 Discussion

The results of MD simulations for a series of systems suggest that the total CN of Na^+^ ions does not depend much on the size of the solvent molecule, salt anion, or salt concentration. On the other hand, the ratio of two types of coordinating oxygen atoms (ether oxygens or oxygen atoms from the anions) changes with the composition of the system. Of course, the amount of anion–cation interactions increases with salt load as a result of increasing number of anion O atoms. At a given concentration, Na^+^ coordination to anions is more probable in the case of TFSI salt and in PEO-based electrolytes. TFSI anions favor bidentate coordination of Na^+^ cations.

In the series of solvents, dynamics of ions becomes slower for longer oligoether chains as a result of increasing viscosity of the electrolyte. Residence times for Na^+^—ether molecules or Na^+^—O_E_ interactions increase in solutions of NaFSI salt. In particular, ether residence times are significantly increased in the 1:6 NaFSI solution in tetraglyme. With the Venn diagrams showing large probability of multiple Na–O interactions between the tetraglyme molecule and metal cation, these results suggest that relatively long-living ion–solvent pairs are formed; therefore, the 1:6 NaFSI/tetraglyme electrolyte acquires the properties of a solvate ionic liquid. The stability of the cation–solvent solvation shell decreases the mobility of ions; therefore, diffusion coefficients and the conductivity of this electrolyte are significantly reduced compared to other tetraglyme-based systems.

For a given solvent/salt pair and the typical diffusion-based mechanism of ion transport, there are two opposing factors governing the conductivity dependence on salt concentration: the amount of available charge carriers (ions), which increases with salt load, and their mobility, which decreases with salt concentration, because of increasing viscosity. Therefore, glyme-based electrolytes exhibit a maximum of conductivity at a certain Me/O ratio.^26,29^ We can see this balance in our results: the decrease in conductivity between 1:20 and 1:6 electrolytes is smaller than the decrease in diffusion coefficients. However, at high salt concentration, an alternative mechanism of ion hopping between aggregates may become operative;^29,43^ its effectiveness may be dependent on the solvent.^29^ In this work, we showed that the interplay between ion concentration and mobility may be also affected by the anion of the salt, changing the degree of correlations. Through the analysis of contributions to the total conductivity, we found that ion–ion correlations in NaTFSI solutions are much more important than in NaFSI electrolytes. The amount of correlations may be related to a higher probability of Na^+^—anion interactions in the former system evidenced by CNs. The difference between NaTFSI and NaFSI solutions becomes smaller in PEO systems, where both types of electrolytes exhibit similar conductivity in accordance with measured values.^33^ The Na^+^–anion interaction energies obtained from quantum-chemical calculations are quite similar and depending on the methodology (e.g., MP2 or DFT) either FSI or TFSI is predicted to interact stronger.^68^ Therefore, it is interesting that the experimentally observed differences between electrolytes based on these anions are larger than that it might be expected from computed binding energies, suggesting that the appropriate FF parameterization for these systems may be a nontrivial task.

## 4 Conclusions

We performed a systematic classical MD study of ether-based electrolytes with two sodium salts, NaFSI or NaTFSI, and monoglyme, tetraglyme, or short PEO chains as solvents. Properties of the electrolytes related to the structure and ion transport were analyzed.

CNs and binding patterns of ether molecules differ between systems with FSI and TFSI anions; TFSI anions more frequently interact with Na cations, whereas FSI anions increase Na^+^-ether binding. Dynamic properties (residence times, diffusion, and conductivity) are mainly determined by the length of solvent molecules; nevertheless, differences between systems based on different anions are observed. These two kinds of electrolytes differ in degree of ion–ion correlations, which are larger for NaTFSI, and affect ion-transport properties. Differences are larger for solutions using glyme molecules and decrease in the PEO-based electrolytes.

There are some discrepancies with experimental data, for example, amount of ion aggregates in PEO determined from vibrational spectra; therefore, another systematic study using, for all systems, more advanced MD parameterization, possibly a purposely developed FF trained on ab initio MD results, would be desirable. Another extension of the work would be the application of first-principles MD simulations to perform a study of the effects of ion–ion and ion–solvent interactions in vibrational spectroscopy, as recently reported for Li and Na salts in cyclic carbonates.^69^ These calculations are planned for the future work.

## Supporting Information Available

RDFs for 1:20 electrolytes, histograms of CNs, Venn diagrams for 1:20 electrolytes, distribution of the distance between binding sites in PEO, residence time autocorrelation functions, and parameters of the FF (PDF)

## References

[ref1] Peters J.; Buchholz D.; Passerini S.; Weil M. Life Cycle Assessment of Sodium-Ion Batteries. Energy Environ. Sci. 2016, 9, 1744–1751. 10.1039/c6ee00640j.

[ref2] Palomares V.; Serras P.; Villaluenga I.; Hueso K. B.; Carretero-González J.; Rojo T. Na-Ion Batteries, Recent Advances and Present Challenges to Become Low Cost Energy Storage Systems. Energy Environ. Sci. 2012, 5, 5884–5901. 10.1039/c2ee02781j.

[ref3] Pan H.; Hu Y.-S.; Chen L. Room-Temperature Stationary Sodium-Ion Batteries for Large-Scale Electric Energy Storage. Energy Environ. Sci. 2013, 6, 2338–2360. 10.1039/c3ee40847g.

[ref4] Yabuuchi N.; Kubota K.; Dahbi M.; Komaba S. Research Development on Sodium-Ion Batteries. Chem. Rev. 2014, 114, 11636–11682. 10.1021/cr500192f. 25390643

[ref5] Nayak P. K.; Yang L.; Brehm W.; Adelhelm P. From Lithium-Ion to Sodium-Ion Batteries: Advantages, Challenges, and Surprises. Angew. Chem., Int. Ed. 2018, 57, 102–120. 10.1002/anie.201703772. 28627780

[ref6] Ponrouch A.; Marchante E.; Courty M.; Tarascon J.-M.; Palacín M. R. In Search of an Optimized Electrolyte for Na-Ion Batteries. Energy Environ. Sci. 2012, 5, 8572–8583. 10.1039/c2ee22258b.

[ref7] Ponrouch A.; Dedryvère R.; Monti D.; Demet A. E.; Ateba Mba J. M.; Croguennec L.; Masquelier C.; Johansson P.; Palacín M. R. Towards High Energy Density Sodium Ion Batteries Through Electrolyte Optimization. Energy Environ. Sci. 2013, 6, 2361–2369. 10.1039/c3ee41379a.

[ref8] Mohd Noor S. A.; Howlett P. C.; MacFarlane D. R.; Forsyth M. Properties of Sodium-Based Ionic Liquid Electrolytes for Sodium Secondary Battery Applications. Electrochim. Acta 2013, 114, 766–771. 10.1016/j.electacta.2013.09.115.

[ref9] Monti D.; Jónsson E.; Palacín M. R.; Johansson P. Ionic Liquid Based Electrolytes for Sodium-Ion Batteries: Na^+^ Solvation and Ionic Conductivity. J. Power Sources 2014, 245, 630–636. 10.1016/j.jpowsour.2013.06.153.

[ref10] Ponrouch A.; Monti D.; Boschin A.; Steen B.; Johansson P.; Palacín M. R. Non-Aqueous Electrolytes for Sodium-Ion Batteries. J. Mater. Chem. A 2015, 3, 22–42. 10.1039/c4ta04428b. 33021285

[ref11] Forsyth M.; Yoon H.; Chen F.; Zhu H.; MacFarlane D. R.; Armand M.; Howlett P. C. Novel Na^+^ Ion Diffusion Mechanism in Mixed Organic–Inorganic Ionic Liquid Electrolyte Leading to High Na^+^ Transference Number and Stable, High Rate Electrochemical Cycling of Sodium Cells. J. Phys. Chem. C 2016, 120, 4276–4286. 10.1021/acs.jpcc.5b11746.

[ref12] Chen C.-Y.; Kiko T.; Hosokawa T.; Matsumoto K.; Nohira T.; Hagiwara R. Ionic Liquid Electrolytes with High Sodium Ion Fraction for High-Rate and Long-Life Sodium Secondary Batteries. J. Power Sources 2016, 332, 51–59. 10.1016/j.jpowsour.2016.09.099.

[ref13] Flores E.; Åvall G.; Jeschke S.; Johansson P. Solvation Structure in Dilute to Highly Concentrated Electrolytes for Lithium-Ion and Sodium-Ion Batteries. Electrochim. Acta 2017, 233, 134–141. 10.1016/j.electacta.2017.03.031.

[ref14] Eshetu G. G.; Elia G. A.; Armand M.; Forsyth M.; Komaba S.; Rojo T.; Passerini S. Electrolytes and Interphases in Sodium-Based Rechargeable Batteries: Recent Advances and Perspectives. Adv. Energy Mater. 2020, 10, 2000093 10.1002/aenm.202000093.

[ref15] Chen F.; Howlett P.; Forsyth M. Na-Ion Solvation and High Transference Number in Superconcentrated Ionic Liquid Electrolytes: A Theoretical Approach. J. Phys. Chem. C 2018, 122, 105–114. 10.1021/acs.jpcc.7b09322.

[ref16] Sun J.; O’Dell L. A.; Armand M.; Howlett P. C.; Forsyth M. Anion-Derived Solid-Electrolyte Interphase Enables Long Life Na-Ion Batteries Using Superconcentrated Ionic Liquid Electrolytes. ACS Energy Lett. 2021, 6, 2481–2490. 10.1021/acsenergylett.1c00816.

[ref17] Stettner T.; Huang P.; Goktas M.; Adelhelm P.; Balducci A. Mixtures of Glyme and Aprotic-Protic Ionic Liquids as Electrolytes for Energy Storage Devices. J. Chem. Phys. 2018, 148, 193825 10.1063/1.5013117. 30307197

[ref18] Westman K.; Dugas R.; Jankowski P.; Wieczorek W.; Gachot G.; Morcrette M.; Irisarri E.; Ponrouch A.; Palacín M. R.; Tarascon J.-M.; et al. Diglyme Based Electrolytes for Sodium-Ion Batteries. ACS Appl. Energy Mater. 2018, 1, 2671–2680. 10.1021/acsaem.8b00360.

[ref19] Goktas M.; Bolli C.; Buchheim J.; Berg E. J.; Novák P.; Bonilla F.; Rojo T.; Komaba S.; Kubota K.; Adelhelm P. Stable and Unstable Diglyme-Based Electrolytes for Batteries with Sodium or Graphite as Electrode. ACS Appl. Mater. Interfaces 2019, 11, 32844–32855. 10.1021/acsami.9b06760. 31397560

[ref20] Mandai T.; Nozawa R.; Tsuzuki S.; Yoshida K.; Ueno K.; Dokko K.; Watanabe M. Phase Diagrams and Solvate Structures of Binary Mixtures of Glymes and Na Salts. J. Phys. Chem. B 2013, 117, 15072–15085. 10.1021/jp407582m. 24236499

[ref21] Mandai T.; Yoshida K.; Tsuzuki S.; Nozawa R.; Masu H.; Ueno K.; Dokko K.; Watanabe M. Effect of Ionic Size on Solvate Stability of Glyme-Based Solvate Ionic Liquids. J. Phys. Chem. B 2015, 119, 1523–1534. 10.1021/jp508100s. 25530321

[ref22] Jache B.; Binder J. O.; Abe T.; Adelhelm P. A Comparative Study on the Impact of Different Glymes and their Derivatives as Electrolyte Solvents for Graphite Co-intercalation Electrodes in Lithium-Ion and Sodium-Ion Batteries. Phys. Chem. Chem. Phys. 2016, 18, 14299–14316. 10.1039/c6cp00651e. 27165175

[ref23] Hasa I.; Dou X.; Buchholz D.; Shao-Horn Y.; Hassoun J.; Passerini S.; Scrosati B. A Sodium-Ion Battery Exploiting Layered Oxide Cathode, Graphite Anode and Glyme-Based Electrolyte. J. Power Sources 2016, 310, 26–31. 10.1016/j.jpowsour.2016.01.082.

[ref24] Wahlers J.; Fulfer K. D.; Harding D. P.; Kuroda D. G.; Kumar R.; Jorn R. Solvation Structure and Concentration in Glyme-Based Sodium Electrolytes: A Combined Spectroscopic and Computational Study. J. Phys. Chem. C 2016, 120, 17949–17959. 10.1021/acs.jpcc.6b06160.

[ref25] Tsuzuki S.; Mandai T.; Suzuki S.; Shinoda W.; Nakamura T.; Morishita T.; Ueno K.; Seki S.; Umebayashi Y.; Dokko K.; et al. Effect of the Cation on the Stability of Cation–Glyme Complexes and their Interactions with the [TFSA]^−^ Anion. Phys. Chem. Chem. Phys. 2017, 19, 18262–18272. 10.1039/c7cp02779f. 28696458

[ref26] Goktas M.; Akduman B.; Huang P.; Balducci A.; Adelhelm P. Temperature-Induced Activation of Graphite Co-intercalation Reactions for Glymes and Crown Ethers in Sodium-Ion Batteries. J. Phys. Chem. C 2018, 122, 26816–26824. 10.1021/acs.jpcc.8b07915.

[ref27] Geysens P.; Rangasamy V. S.; Thayumanasundaram S.; Robeyns K.; Van Meervelt L.; Locquet J.-P.; Fransaer J.; Binnemans K. Solvation Structure of Sodium Bis(fluorosulfonyl)imide-Glyme Solvate Ionic Liquids and Its Influence on Cycling of Na-MNC Cathodes. J. Phys. Chem. B 2018, 122, 275–289. 10.1021/acs.jpcb.7b10158. 29200299

[ref28] Terada S.; Susa H.; Tsuzuki S.; Mandai T.; Ueno K.; Dokko K.; Watanabe M. Glyme–Sodium Bis(fluorosulfonyl)amide Complex Electrolytes for Sodium Ion Batteries. J. Phys. Chem. C 2018, 122, 16589–16599. 10.1021/acs.jpcc.8b04367.

[ref29] Galle Kankanamge S. R.; Li K.; Fulfer K. D.; Du P.; Jorn R.; Kumar R.; Kuroda D. G. Mechanism behind the Unusually High Conductivities of High Concentrated Sodium Ion Glyme-Based Electrolytes. J. Phys. Chem. C 2018, 122, 25237–25246. 10.1021/acs.jpcc.8b06991.

[ref30] Liyana-Arachchi T. P.; Haskins J. B.; Burke C. M.; Diederichsen K. M.; McCloskey B. D.; Lawson J. W. Polarizable Molecular Dynamics and Experiments of 1,2-Dimethoxyethane Electrolytes with Lithium and Sodium Salts: Structure and Transport Properties. J. Phys. Chem. B 2018, 122, 8548–8559. 10.1021/acs.jpcb.8b03445. 30130409

[ref31] Garcia-Quintana L.; Chen F.; Ortiz-Vitoriano N.; Zhang Y.; O’Dell L. A.; MacFarlane D. R.; Forsyth M.; Bond A. M.; Howlett P. C.; Pozo-Gonzalo C. Unravelling the Role of Speciation in Glyme:Ionic Liquid Hybrid Electrolytes for Na-O_2_ Batteries. Batteries Supercaps 2021, 4, 513–521. 10.1002/batt.202000261.

[ref32] Serra Moreno J.; Armand M.; Berman M. B.; Greenbaum S. G.; Scrosati B.; Panero S. Composite PEO_n_:NaTFSI polymer electrolyte: Preparation, thermal and electrochemical characterization. J. Power Sources 2014, 248, 695–702. 10.1016/j.jpowsour.2013.09.137.

[ref33] Boschin A.; Johansson P. Characterization of NaX (X: TFSI, FSI) – PEO based solid polymer electrolytes for sodium batteries. Electrochim. Acta 2015, 175, 124–133. 10.1016/j.electacta.2015.03.228.

[ref34] Boschin A.; Johansson P. Plasticization of NaX-PEO solid polymer electrolytes by Pyr_13_X ionic liquids. Electrochim. Acta 2016, 211, 1006–1015. 10.1016/j.electacta.2016.06.119.

[ref35] Yang Y. Q.; Chang Z.; Li M. X.; Wang X. W.; Wu Y. P. A sodium ion conducting gel polymer electrolyte. Solid State Ionics 2015, 269, 1–7. 10.1016/j.ssi.2014.11.015.

[ref36] Forsyth M.; Porcarelli L.; Wang X.; Goujon N.; Mecerreyes D. Innovative Electrolytes Based on Ionic Liquids and Polymers for Next-Generation Solid-State Batteries. Acc. Chem. Res. 2019, 52, 686–694. 10.1021/acs.accounts.8b00566. 30801170

[ref37] Dong H.; Hyun J.-K.; Rhodes C. P.; Frech R.; Wheeler R. A. Molecular Dynamics Simulations and Vibrational Spectroscopic Studies of Local Structure in Tetraglyme:Sodium Triflate (CH_3_O(CH_2_CH_2_O)_4_CH_3_:NaCF_3_SO_3_) Solutions. J. Phys. Chem. B 2002, 106, 4878–4885. 10.1021/jp013914w.

[ref38] Liu Q.; Wu F.; Mu D.; Wu B. A Theoretical Study on Na^+^ Solvation in Carbonate Ester and Ether Solvents for Sodium-ion Batteries. Phys. Chem. Chem. Phys. 2020, 22, 2164–2175. 10.1039/c9cp05636j. 31912812

[ref39] Payne V. A.; Xu J.-H.; Forsyth M.; Ratner M. A.; Shriver D. F.; de Leeuw S. W. Ion Clustering in Molecular Dynamics Simulations of Sodium Iodide Solutions. Electrochim. Acta 1995, 40, 2087–2091. 10.1016/0013-4686(95)00145-5.

[ref40] Payne V. A.; Xu J. h.; Forsyth M.; Ratner M. A.; Shriver D. F.; de Leeuw S. W. Molecular Dynamics Simulations of Ion Clustering and Conductivity in NaI/Ether Solutions. Effect of Ion Concentration. J. Chem. Phys. 1995, 103, 8746–8755. 10.1063/1.470131.

[ref41] Neyertz S.; Brown D.; Thomas J. O. Molecular Dynamics Simulation of the Crystalline Phase of Poly(Ethylene Oxide)-Sodium Iodide, PEO3NaI. Electrochim. Acta 1995, 40, 2063–2069. 10.1016/0013-4686(95)91266-b.

[ref42] Neyertz S.; Brown D. Phase Separation upon Heating in Model PEO_x_NaI Polymer Electrolytes. Electrochim. Acta 1998, 43, 1343–1347. 10.1016/s0013-4686(97)10041-x.

[ref43] Okoshi M.; Chou C.-P.; Nakai H. Theoretical Analysis of Carrier Ion Diffusion in Superconcentrated Electrolyte Solutions for Sodium-Ion Batteries. J. Phys. Chem. B 2018, 122, 2600–2609. 10.1021/acs.jpcb.7b10589. 29433319

[ref44] Åvall G.; Mindemark J.; Brandell D.; Johansson P. Sodium-Ion Battery Electrolytes: Modeling and Simulations. Adv. Energy Mater. 2018, 8, 1703036 10.1002/aenm.201703036.

[ref45] Åvall G.; Johansson P. A Novel Approach to Ligand-Exchange Rates Applied to Lithium-Ion Battery and Sodium-Ion Battery Electrolytes. J. Chem. Phys. 2020, 152, 234104 10.1063/5.0005397. 32571038

[ref46] Martínez L.; Andrade R.; Birgin E. G.; Martínez J. M. Packmol: A Package for Building Initial Configurations for Molecular Dynamics Simulations. J. Comput. Chem. 2009, 30, 2157–2164. 10.1002/jcc.21224. 19229944

[ref47] SCIGRESS; FQS Poland Ltd.: Kraków, Poland, https://www.fqs.pl/en/chemistry/products/scigress.

[ref48] Lamoureux G.; Roux B. Modeling Induced Polarization with Classical Drude Oscillators: Theory and Molecular Dynamics Simulation Algorithm. J. Chem. Phys. 2003, 119, 3025–3039. 10.1063/1.1589749.

[ref49] Anderson P. M.; Wilson M. R. Developing a Force Field for Simulation of Poly(ethylene oxide) Based upon ab Initio Calculations of 1,2-Dimethoxyethane. Mol. Phys. 2005, 103, 89–97. 10.1080/00268970412331293811.

[ref50] Wang J.; Wang W.; Kollman P. A.; Case D. A. Automatic Atom Type and Bond Type Perception in Molecular Mechanical Calculations. J. Mol. Graphics Modell. 2006, 25, 247–260. 10.1016/j.jmgm.2005.12.005. 16458552

[ref51] Wang J.; Wolf R. M.; Caldwell J. W.; Kollman P. A.; Case D. A. Development and Testing of a General AMBER Force Field. J. Comput. Chem. 2004, 25, 1157–1174. 10.1002/jcc.20035. 15116359

[ref52] Cornell W. D.; Cieplak P.; Bayly C. I.; Gould I. R.; Merz K. M. Jr.; Ferguson D. M.; Spellmeyer D. C.; Fox T.; Caldwell J. W.; Kollman P. A. Second Generation Force Field for the Simulation of Proteins, Nucleic Acids, and Organic Molecules. J. Am. Chem. Soc. 1995, 117, 5179–5197. 10.1021/ja00124a002.

[ref53] Borodin O.; Smith G. D. Development of Quantum Chemistry-Based Force Fields for Poly(ethylene oxide) with Many-Body Polarization Interactions. J. Phys. Chem. B 2003, 107, 6801–6812. 10.1021/jp027537e.

[ref54] Canongia Lopes J. N.; Pádua A. A. H. Molecular Force Field for Ionic Liquids Composed of Triflate or Bistriflylimide Anions. J. Phys. Chem. B 2004, 108, 16893–16898. 10.1021/jp0476545.

[ref55] Shimizu K.; Almantariotis D.; Gomes M. F. C.; Pádua A. A. H.; Canongia Lopes J. N. Molecular Force Field for Ionic Liquids V: Hydroxyethylimidazolium, Dimethoxy-2- Methylimidazolium, and Fluoroalkylimidazolium Cations and Bi (Fluorosulfonyl)Amide, Perfluoroalkanesulfonylamide, and Fluoroalkylfluorophosphate Anions. J. Phys. Chem. B 2010, 114, 3592–3600. 10.1021/jp9120468. 20175555

[ref56] Borodin O. Polarizable Force Field Development and Molecular Dynamics Simulations of Ionic Liquids. J. Phys. Chem. B 2009, 113, 11463–11478. 10.1021/jp905220k. 19637900

[ref57] Köddermann T.; Paschek D.; Ludwig R. Molecular Dynamic Simulations of Ionic Liquids: A Reliable Description of Structure, Thermodynamics and Dynamics. ChemPhysChem 2007, 8, 2464–2470. 10.1002/cphc.200700552. 17943710

[ref58] Jensen K. P.; Jorgensen W. L. Halide, Ammonium, and Alkali Metal Ion Parameters for Modeling Aqueous Solutions. J. Chem. Theory Comput. 2006, 2, 1499–1509. 10.1021/ct600252r. 26627020

[ref59] Phillips J. C.; Braun R.; Wang W.; Gumbart J.; Tajkhorshid E.; Villa E.; Chipot C.; Skeel R. D.; Kalé L.; Schulten K. Scalable Molecular Dynamics with NAMD. J. Comput. Chem. 2005, 26, 1781–1802. 10.1002/jcc.20289. 16222654 PMC2486339

[ref60] Feller S. E.; Zhang Y.; Pastor R. W.; Brooks B. R. Constant Pressure Molecular Dynamics Simulation: The Langevin Piston Method. J. Chem. Phys. 1995, 103, 4613–4621. 10.1063/1.470648.

[ref61] Martyna G. J.; Tobias D. J.; Klein M. L. Constant Pressure Molecular Dynamics Algorithms. J. Chem. Phys. 1994, 101, 4177–4189. 10.1063/1.467468.

[ref62] Darden T.; York D.; Pedersen L. Particle Mesh Ewald: An Nlog(N) Method for Ewald Sums in Large Systems. J. Chem. Phys. 1993, 98, 10089–10092. 10.1063/1.464397.

[ref63] Monti D.; Jónsson E.; Boschin A.; Palacín M. R.; Ponrouch A.; Johansson P. Towards Standard Electrolytes for Sodium-Ion Batteries: Physical Properties, Ion Solvation and Ion-Pairing in Alkyl Carbonate Solvents. Phys. Chem. Chem. Phys. 2020, 22, 22768–22777. 10.1039/d0cp03639k. 33021285

[ref64] Monti D.; Ponrouch A.; Palacín M. R.; Johansson P. Towards Safer Sodium-Ion Batteries via Organic Solvent/Ionic Liquid Based Hybrid Electrolytes. J. Power Sources 2016, 324, 712–721. 10.1016/j.jpowsour.2016.06.003.

[ref65] Chen X.; Chen F.; Liu M. S.; Forsyth M. Polymer Architecture Effect on Sodium Ion Transport in PSTFSI-based Ionomers: A Molecular Dynamics Study. Solid State Ionics 2016, 288, 271–276. 10.1016/j.ssi.2015.12.004.

[ref66] Thum A.; Heuer A.; Shimizu K.; Canongia Lopes J. N. Solvate Ionic Liquids Based on Lithium Bis(trifluoromethanesulfonyl)imide–Glyme Systems: Coordination in MD Simulations with Scaled Charges. Phys. Chem. Chem. Phys. 2020, 22, 525–535. 10.1039/c9cp04947a. 31829360

[ref67] Wiencierz M.; Stolwijk N. A. Systematics of Ionic Transport and Pair Formation in Amorphous PEO-NaI Polymer Electrolytes. Solid State Ionics 2012, 212, 88–99. 10.1016/j.ssi.2012.02.002.

[ref68] Jónsson E.; Johansson P. Modern Battery Electrolytes: Ion–Ion Interactions in Li^+^/Na^+^ Conductors from DFT Calculations. Phys. Chem. Chem. Phys. 2012, 14, 10774–10779. 10.1039/c2cp40612h. 22751486

[ref69] Wróbel P.; Kubisiak P.; Eilmes A. MeTFSI (Me = Li, Na) Solvation in Ethylene Carbonate and Fluorinated Ethylene Carbonate: A Molecular Dynamics Study. J. Phys. Chem. B 2021, 125, 1248–1258. 10.1021/acs.jpcb.0c10622. 33482689

