# Supplementary material for: NaFSI and NaTFSI Solutions in Ether Solvents from Monoglyme to Poly(ethylene oxide)—A Molecular Dynamics Study
Source: J Phys Chem B. 2021 Sep 8;125(36):10293–303. doi: 10.1021/acs.jpcb.1c05793 (PMC8450900; doi:10.1021/acs.jpcb.1c05793)
Supplement: Supplementary file 1 — jp1c05793_si_001.pdf [file jp1c05793_si_001.pdf]

NaFSI and NaTFSI Solutions in Ether Solvents from Monoglyme to Poly(ethylene oxide) – a Molecular Dynamics Study

Piotr Wróbel, Piotr Kubisiak, Andrzej Eilmes*

Faculty of Chemistry, Jagiellonian University, Gronostajowa 2, 30-387 Kraków, Poland
eilmes@chemia.uj.edu.pl

Supporting Information

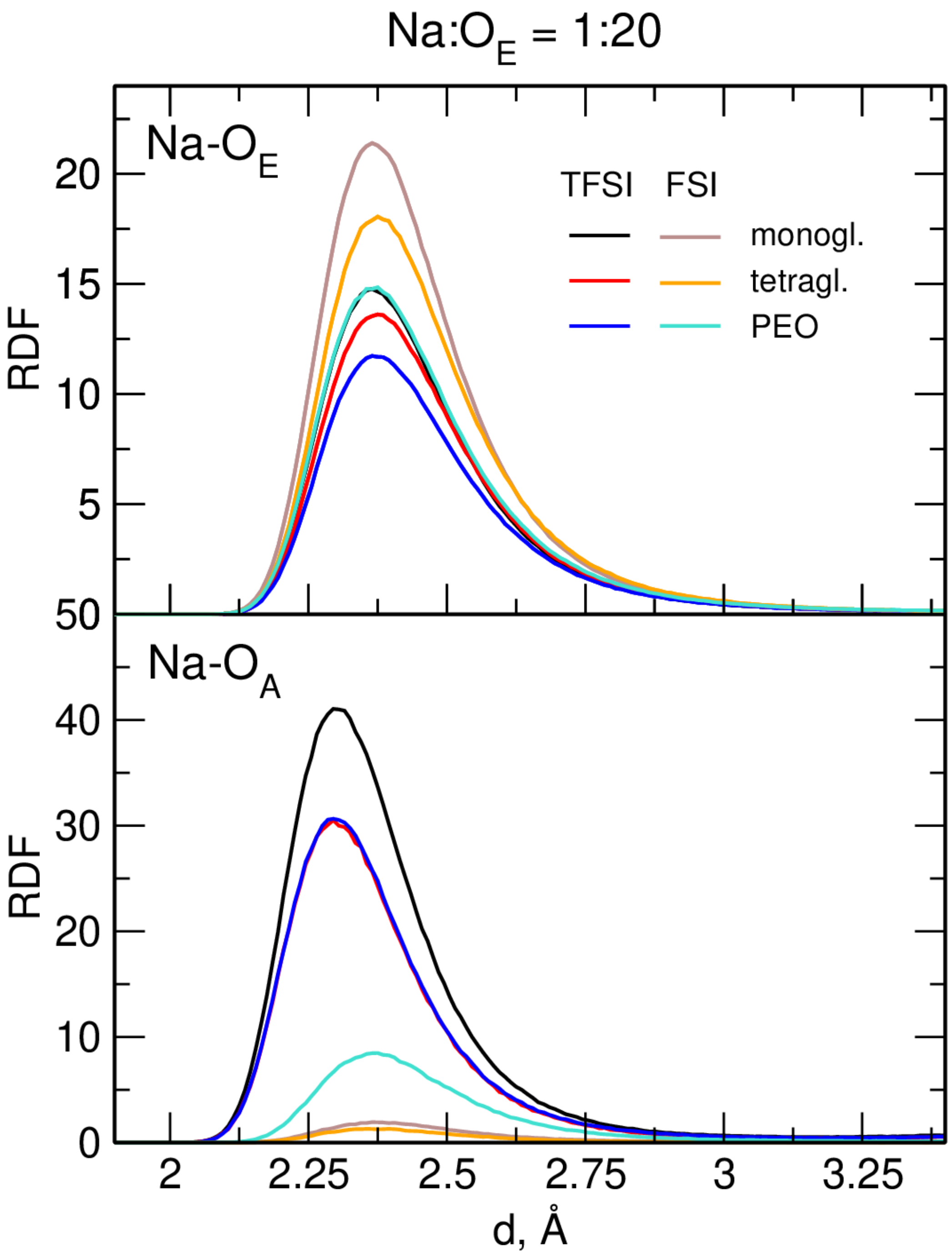

Figure S1. RDFs for Na-O atom pairs in the 1:20 electrolytes.

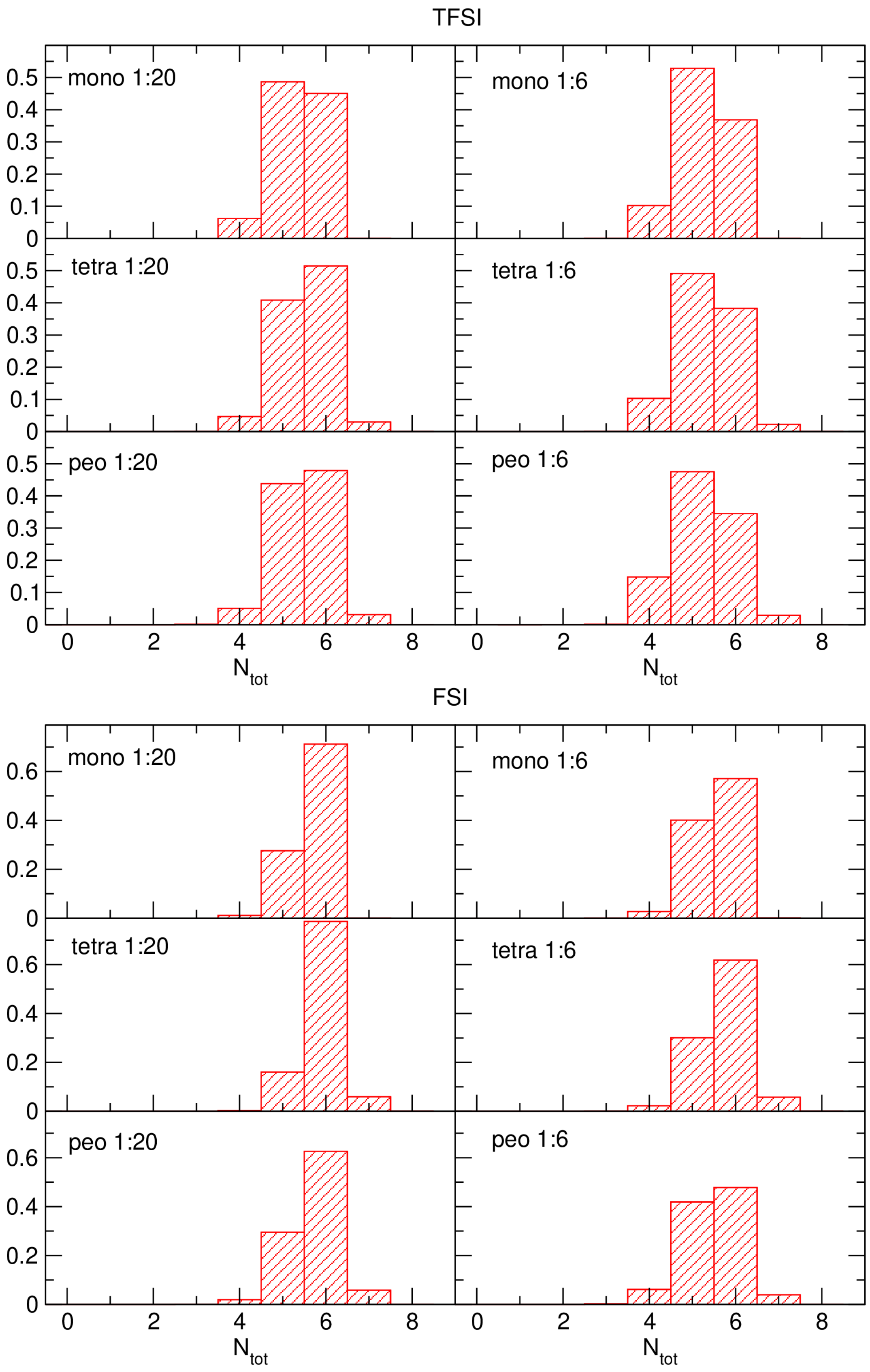

Figure S2. Distributions of the total number of oxygen atoms coordinated to Na^+.

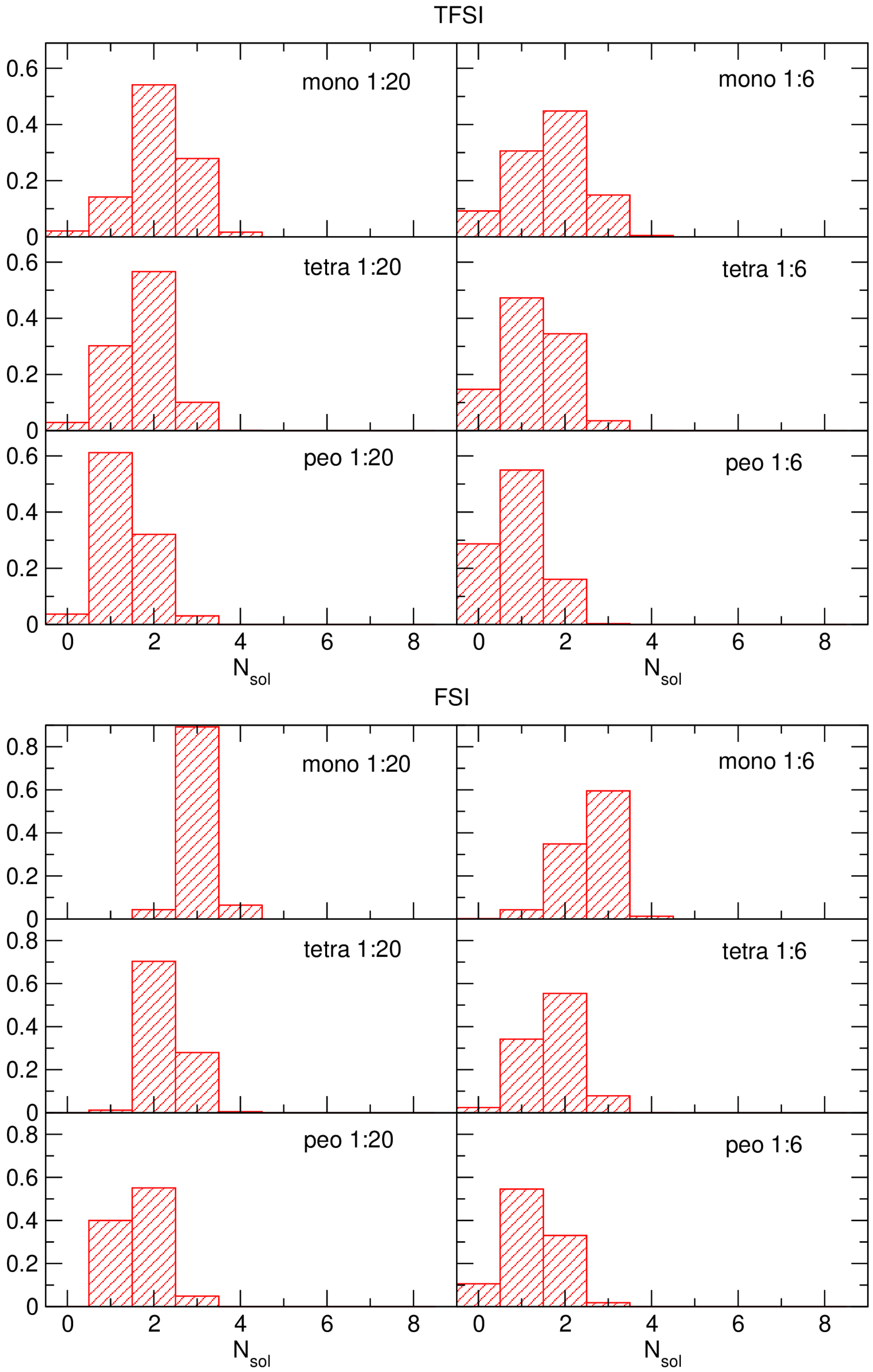

Figure S3. Distributions of the total number of solvent molecules coordinated to Na^+ (solvent numbers, SNs).

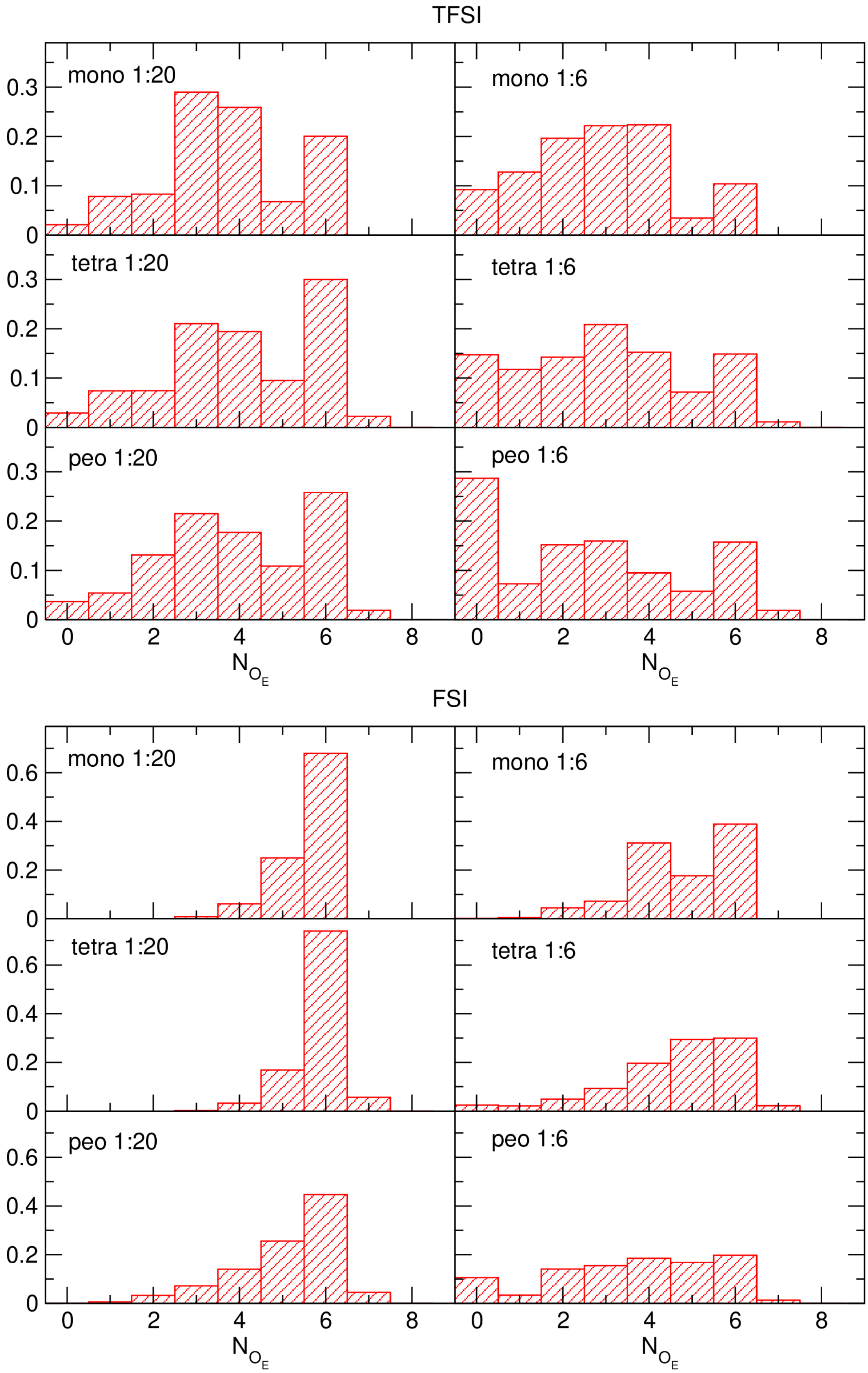

Figure S4. Distributions of the number of ether oxygen atoms coordinated to Na^{+}.

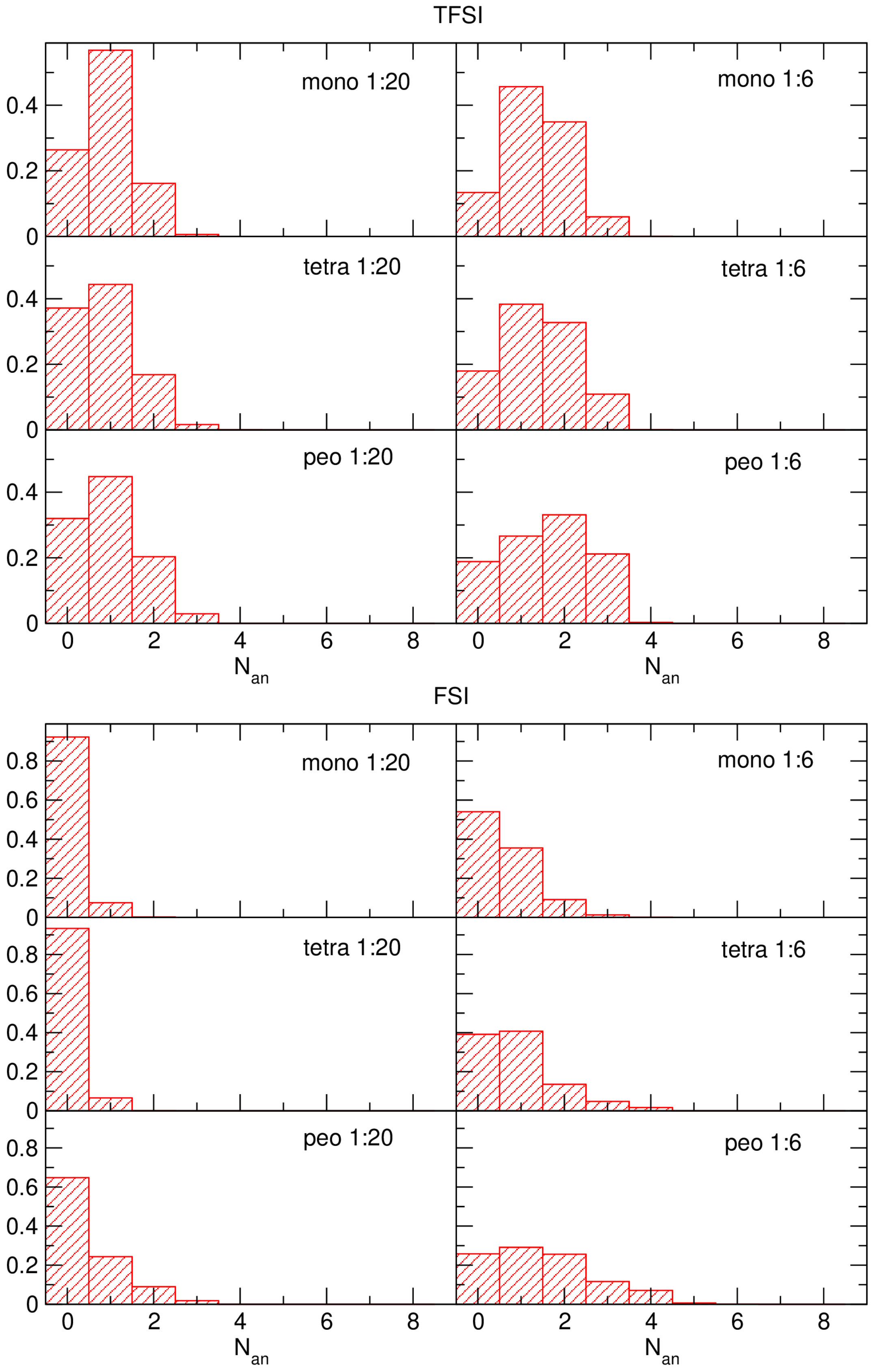

Figure S5. Distributions of the number of anions coordinated to Na^{+}.

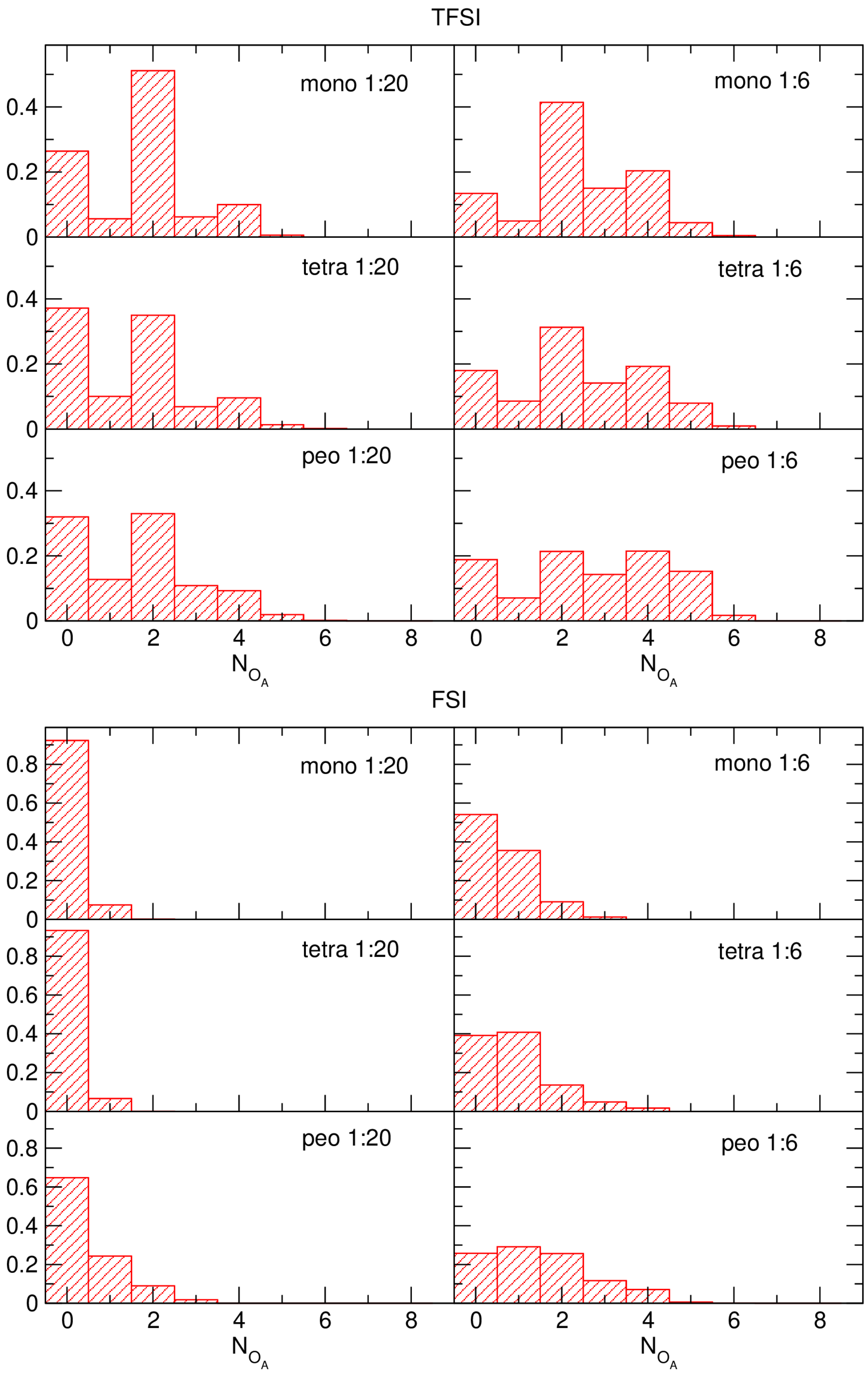

Figure S6. Distributions of the number of anion oxygen atoms coordinated to Na^+.

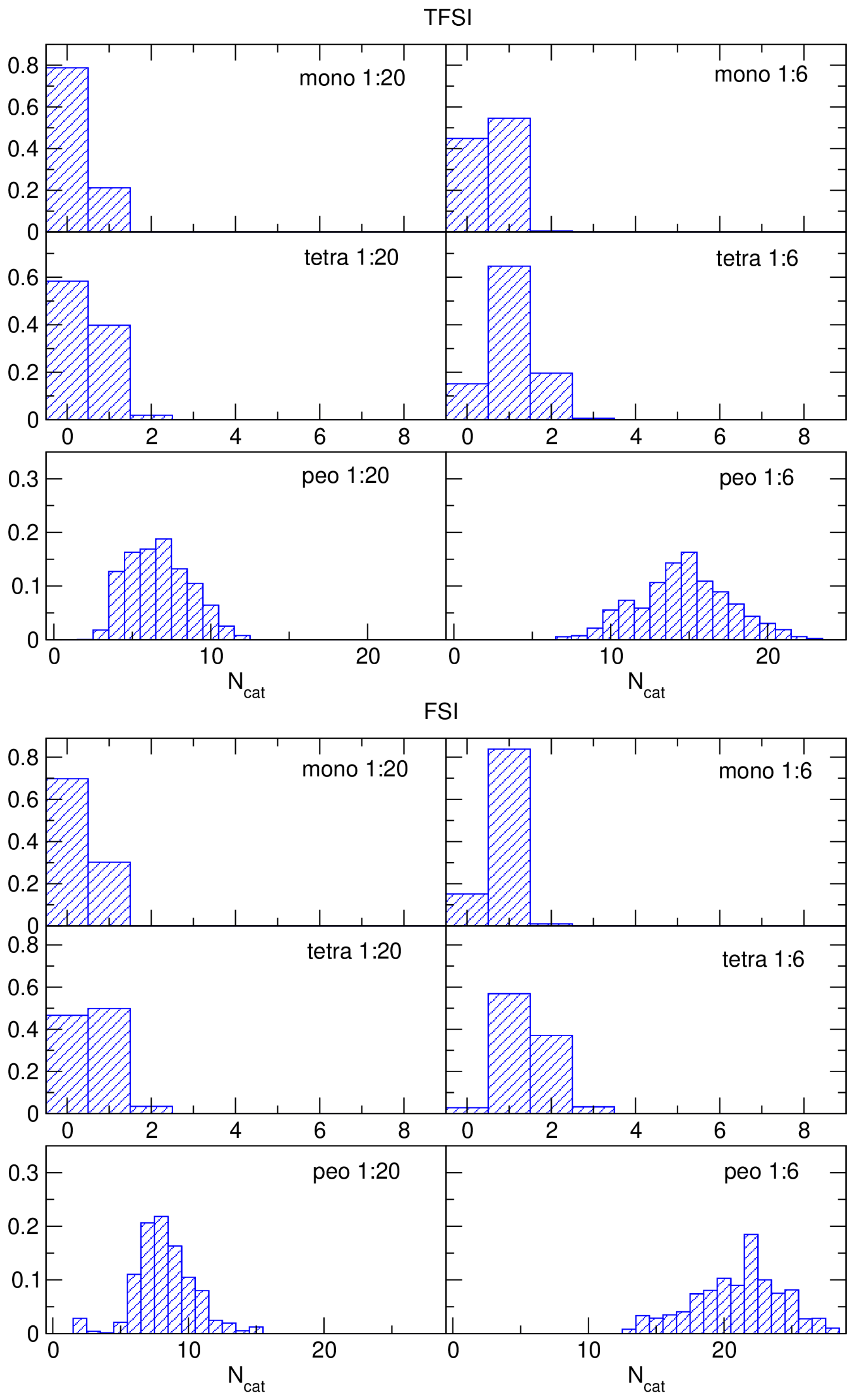

Figure S7. Distributions of the number of Na^+ ions coordinated to solvent molecule

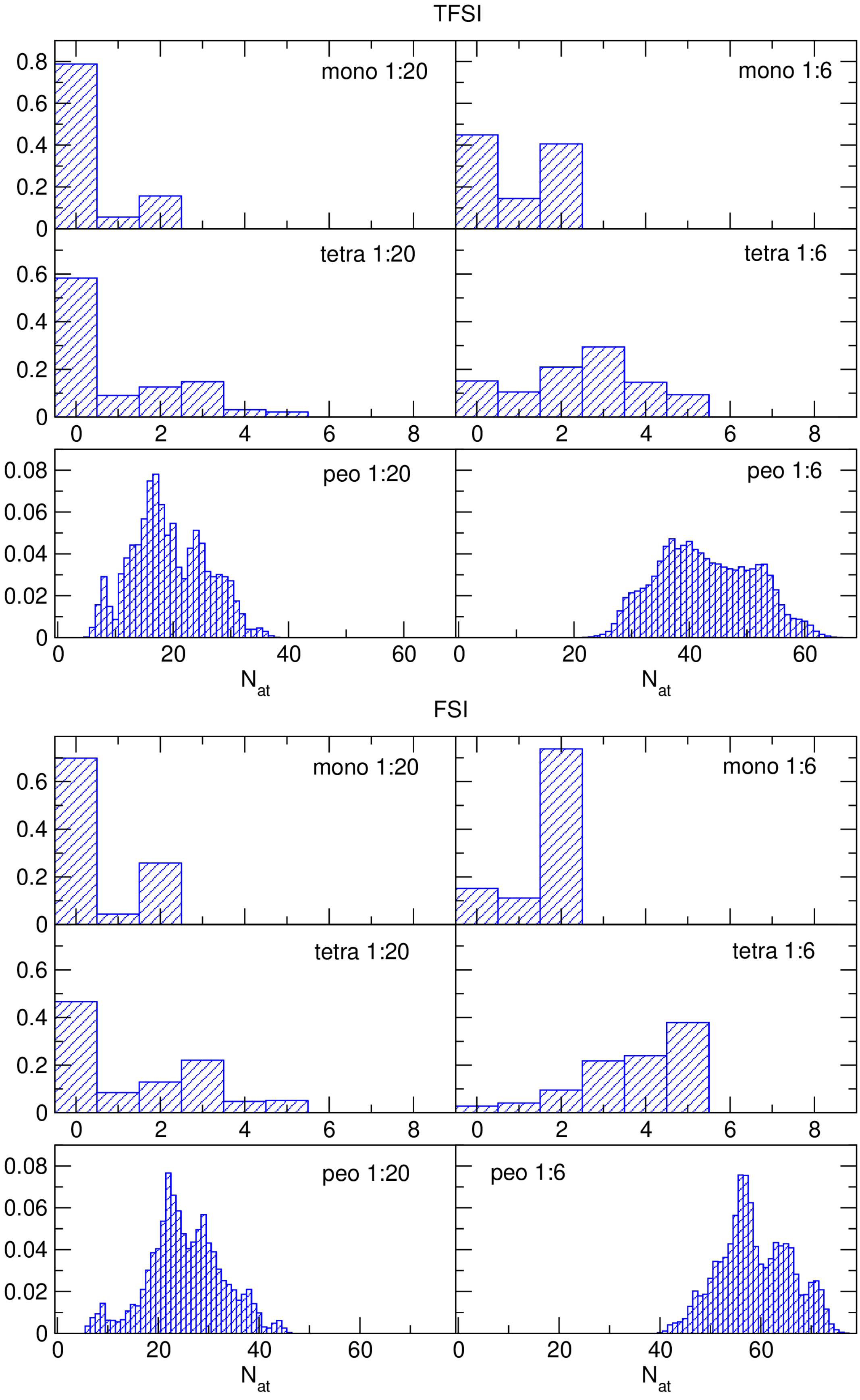

Figure S8. Distributions of the number of oxygen atoms of the solvent molecule engaged in coordination of Na^+ ions.

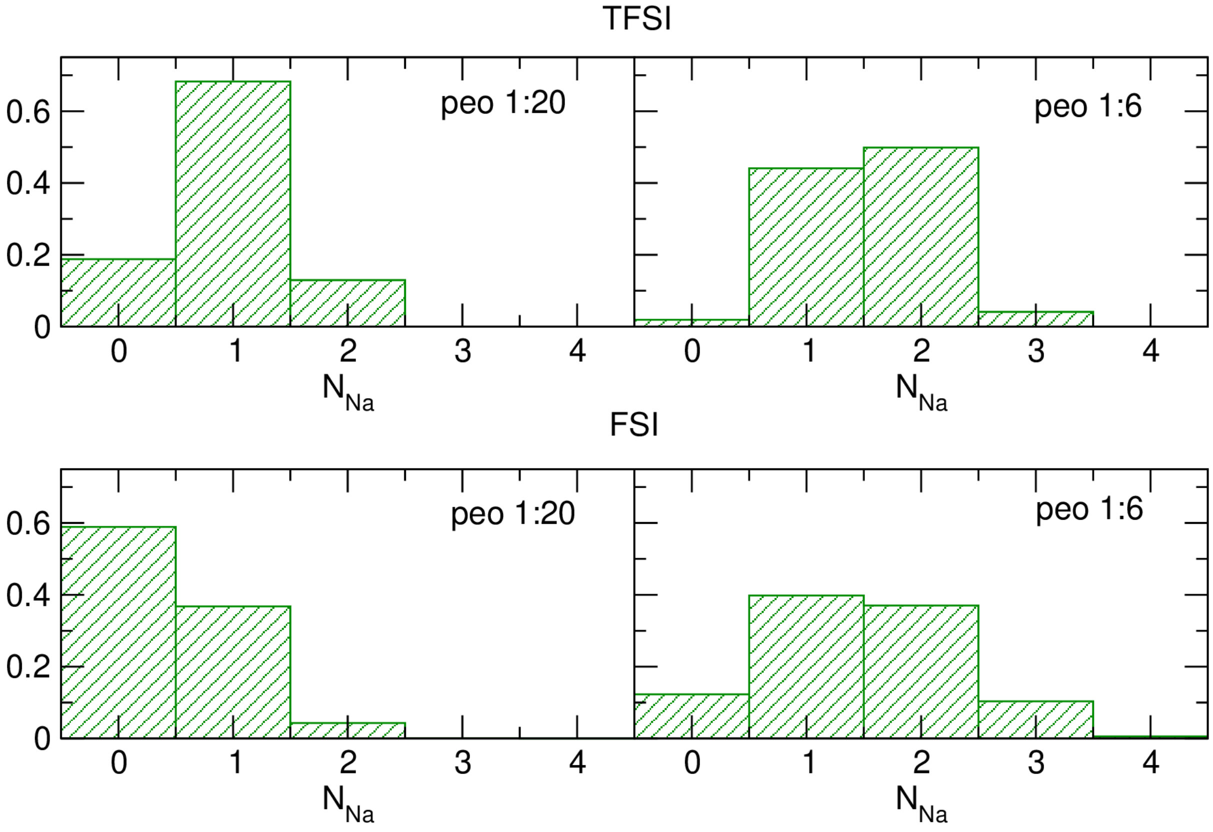

Figure S9. Distributions of the number of Na^+ ions coordinated to anions in PEO electrolytes.

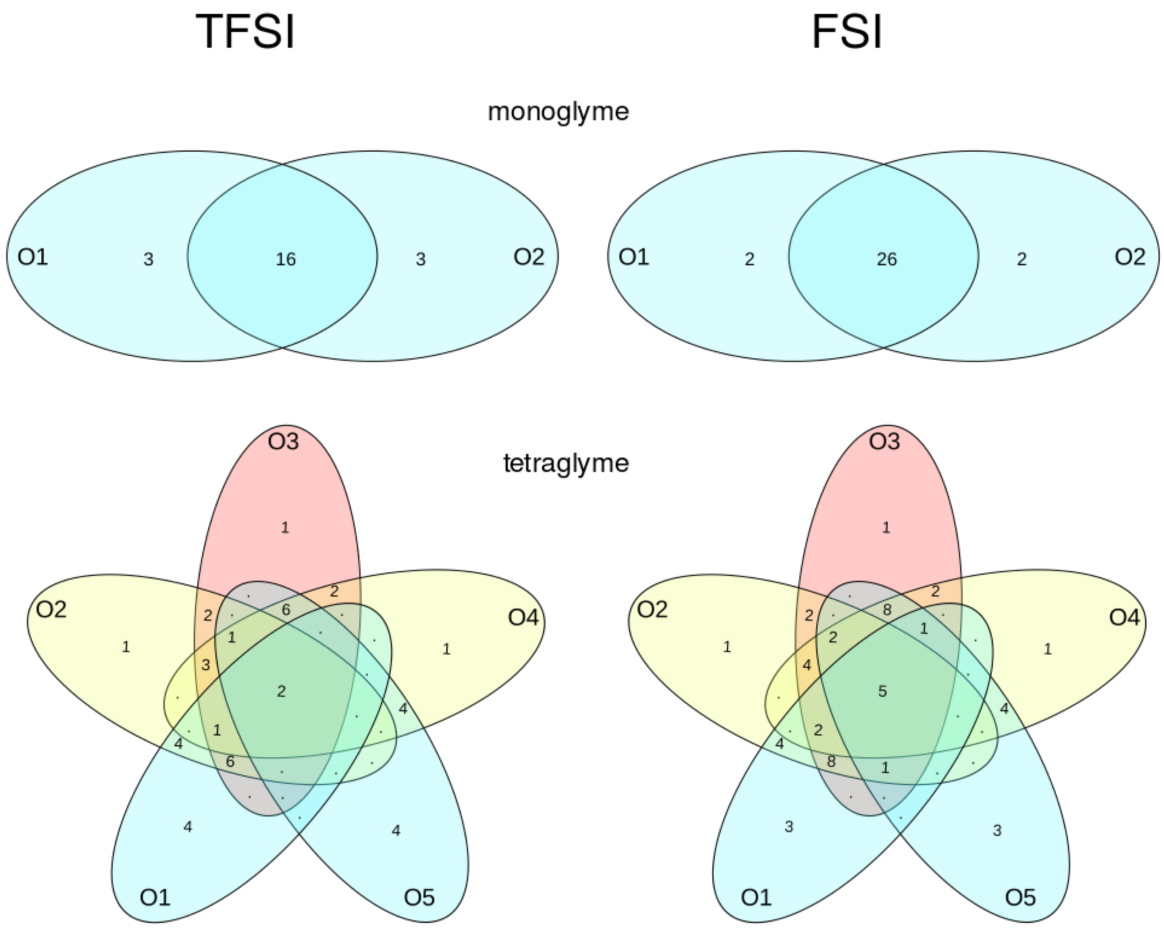

Figure S10. Venn diagrams showing the connectivity between the Na^{+} ion and the O atoms of the monoglyme and tetraglyme molecules in 1:20 electrolytes. Values lower than 1% are displayes as dots.

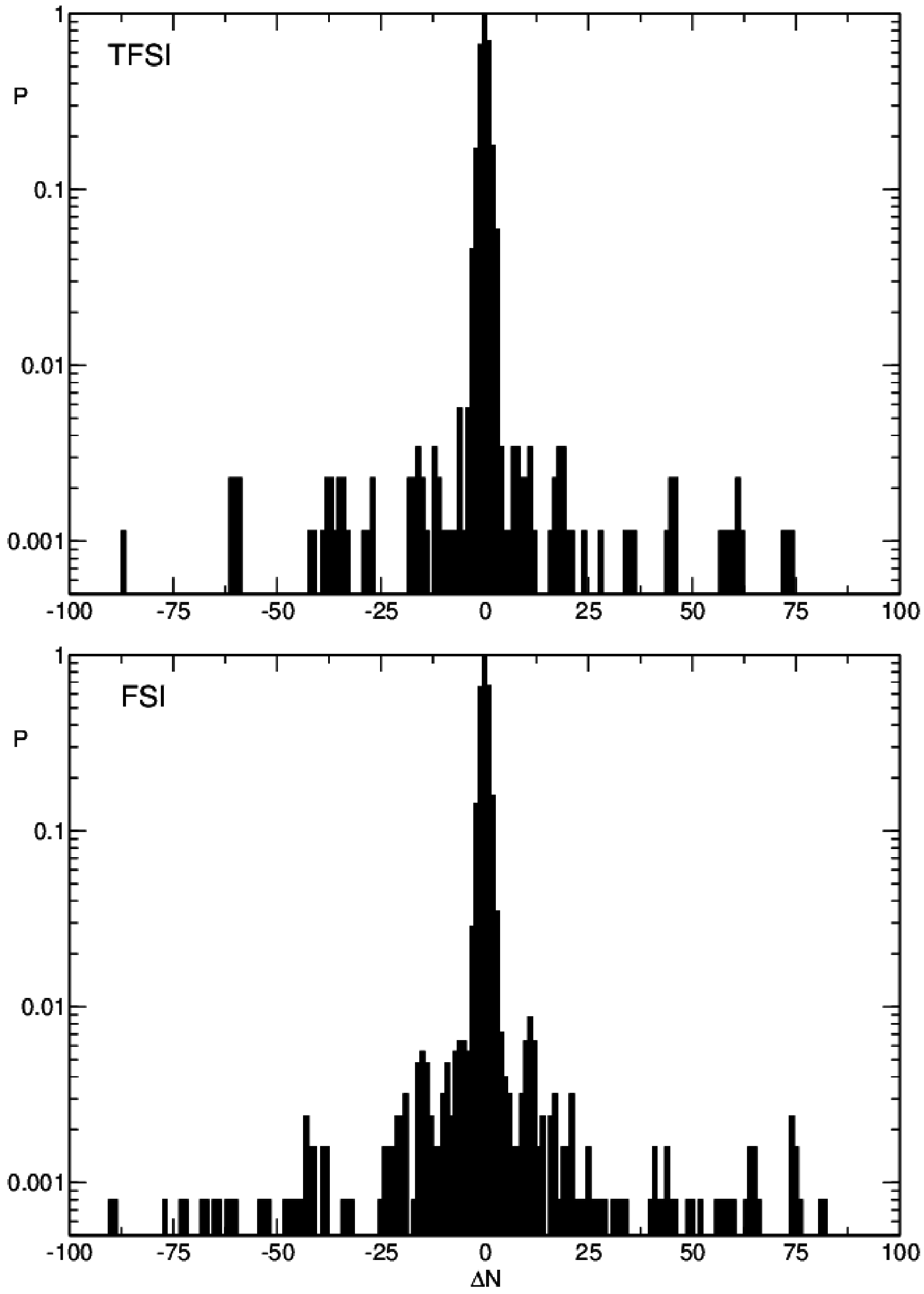

Figure S11. Distribution of the probability that an oxygen atom positioned at the site Δ*N*, is coordinated to the Na^+ cation, interacting with the oxygen atom at the site *N* = 0 in the 1:6 PEO-based electrolytes.

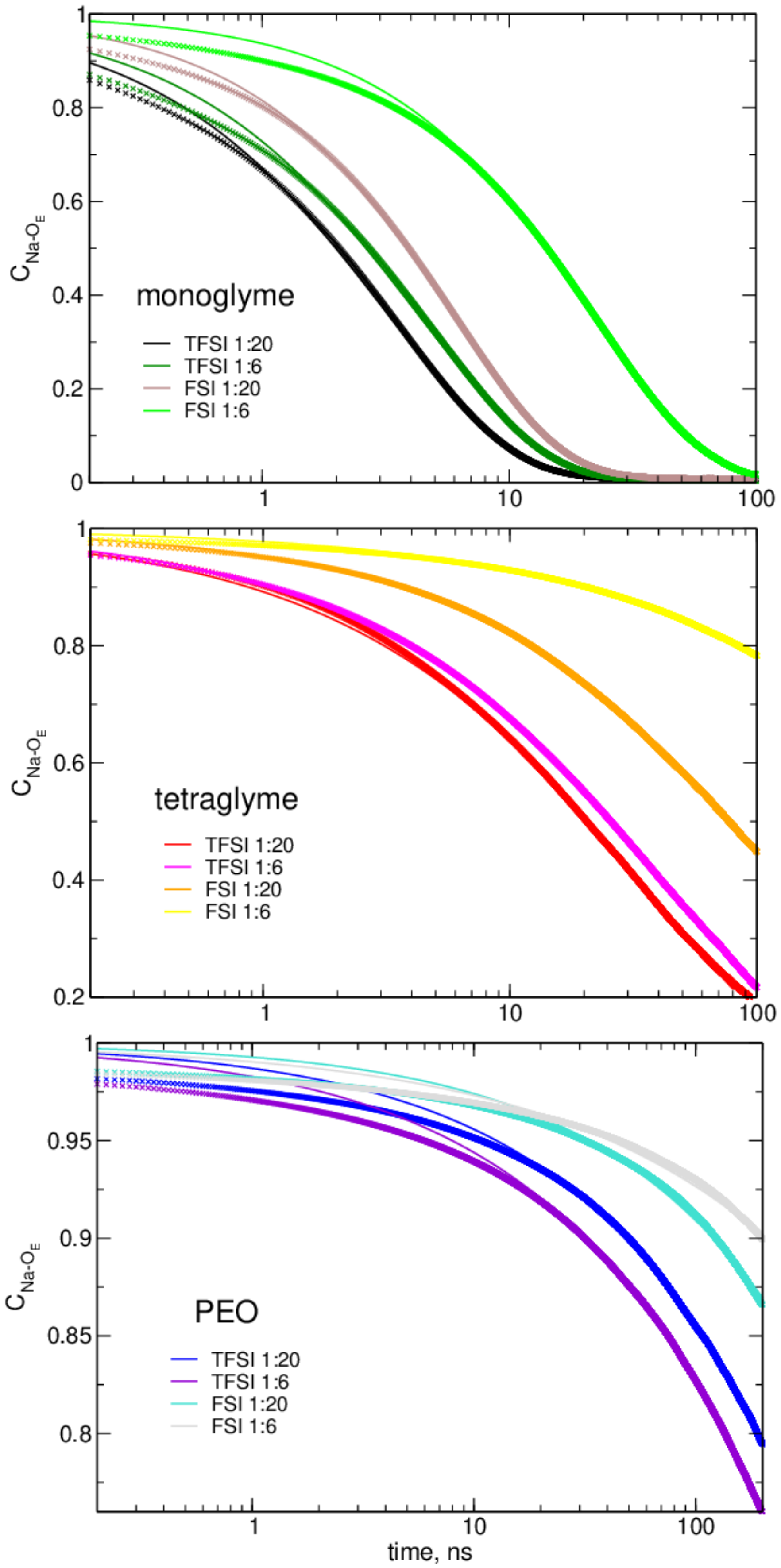

Figure S12. Na-O_E residence time autocorrelation function. Lines are fits to the data.

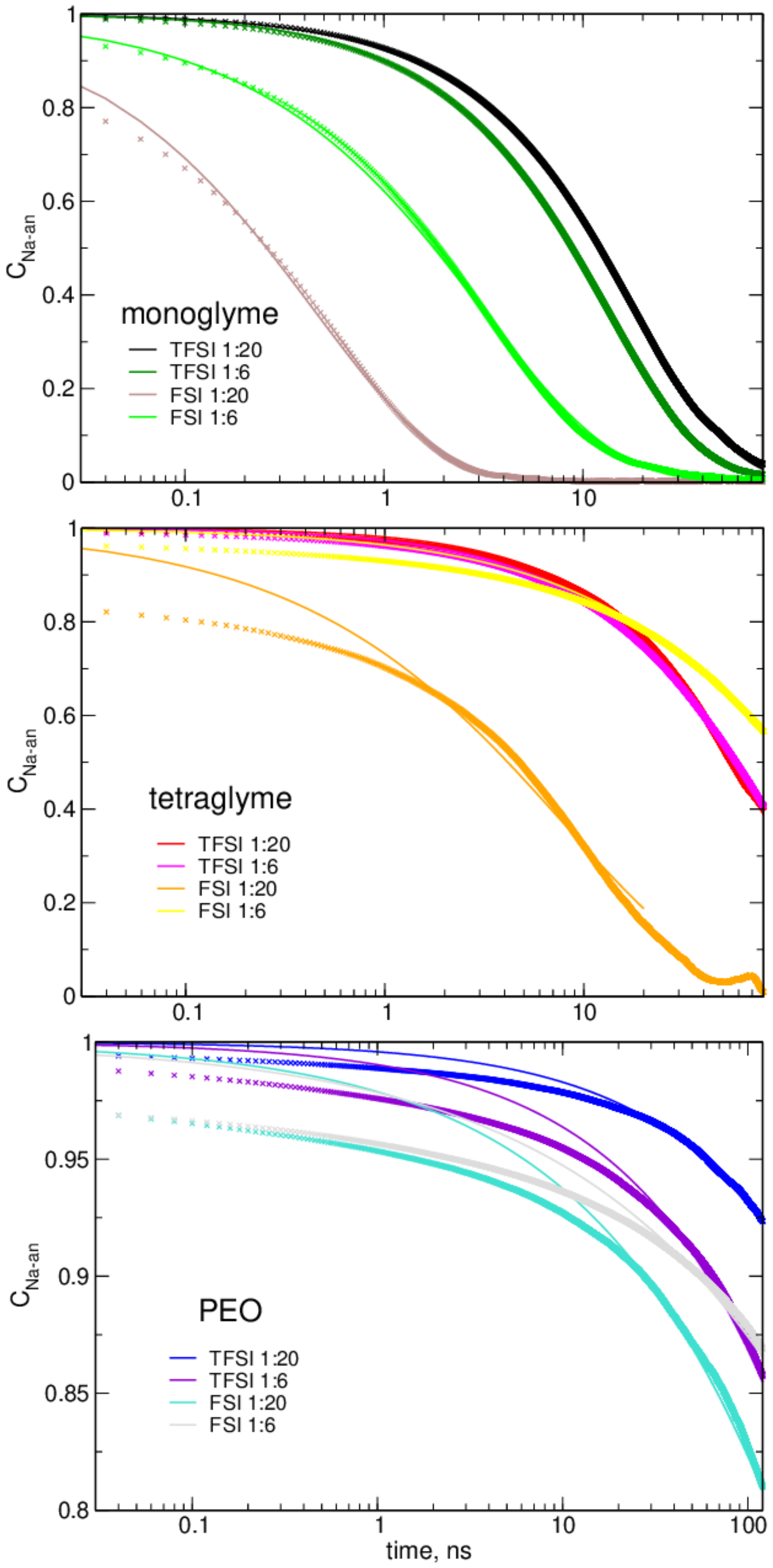

Figure S13. Na-anion residence time autocorrelation function. Lines are fits to the data.

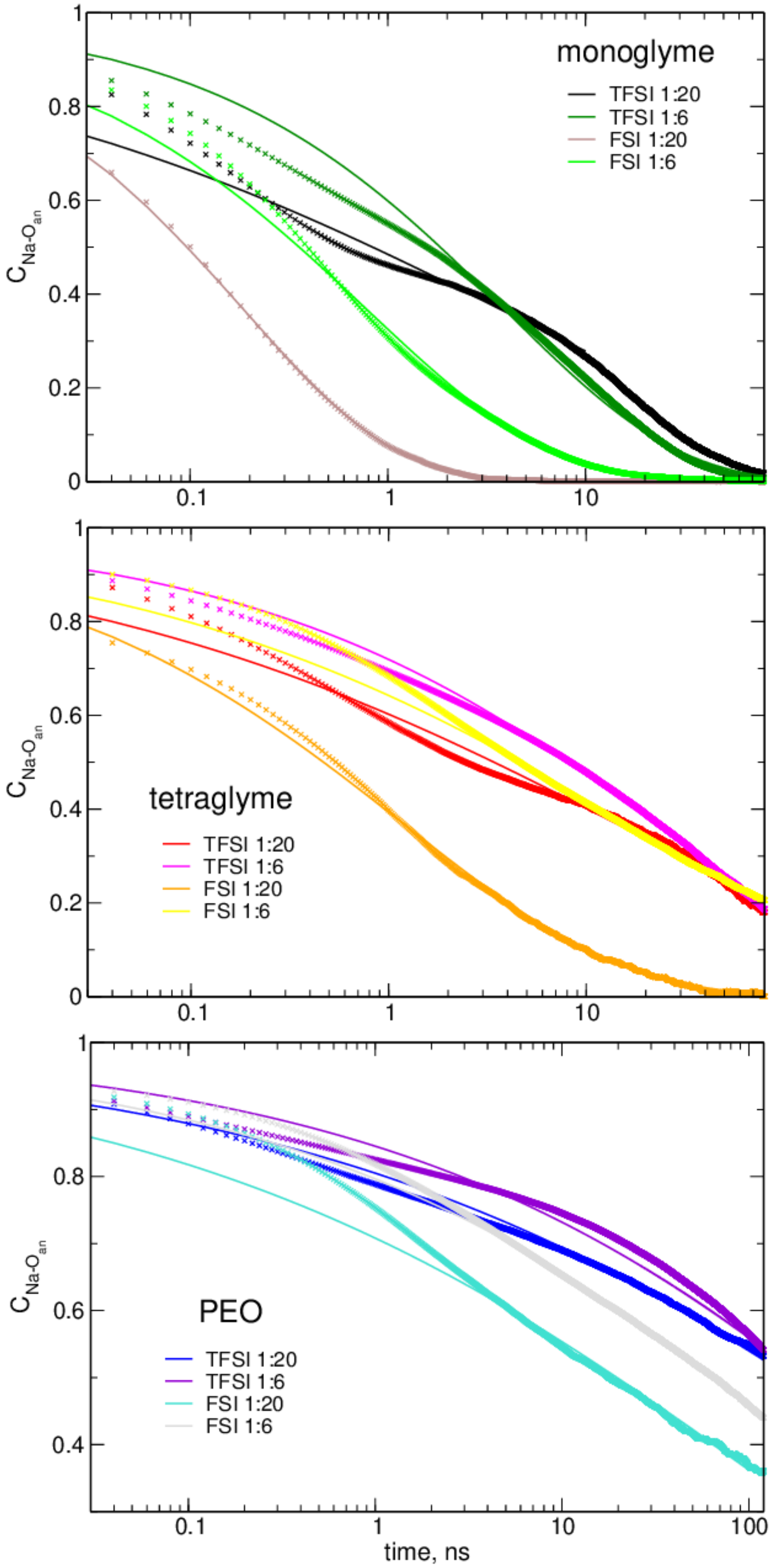

Figure S14. Na-O_A residence time autocorrelation function. Lines are fits to the data.

Appendix

Parameters of the classical FF

$$U_{ij} = \sum_i K_{r,i}\left(r_i - r_{0,i}\right)^2 + \sum_i K_{\theta,i}\left(\theta_i - \theta_{0,i}\right)^2 + \sum_n K_{\xi,n}(1 + \cos(n(\xi) - \gamma)) + \sum_i \sum_{j\neq i}\left\{\varepsilon_{ij}\left(\frac{r_{min,ij}}{r_{ij}}\right)^{12} - 2\left(\frac{r_{min,ij}}{r_{ij}}\right)^{6} + \frac{q_i q_j}{4\pi\varepsilon_0 r_{ij}}\right\}$$

$$\varepsilon_{ij} = \sqrt{\varepsilon_i \varepsilon_j}$$

$$r_{min,ij} = \left(r_{min,i} + r_{min,j}\right)/2$$

Drude particle with partial charge $q_{D,i}$ is attached to atom with polarizability α_i:

$$\alpha_i = \frac{q_{D,i}^2}{2k_D}$$

Partial charge on the parent atom is modified, so that the atom and the Drude particle together carry the original partial charge q of the parent atom.

TFSI/FSI anions

BONDS	K_r [kcal/mole/Å^2]	r_0 [Å]
Ct-Ft	441.8	1.323
St-Ct	235.4	1.818
St-Ot	637.1	1.442
Nt-St	372.0	1.570
Nf-Sf	374.9	1.570
Sf-Of	637.1	1.437
Sf-Ff	224.5	1.575

TFSI

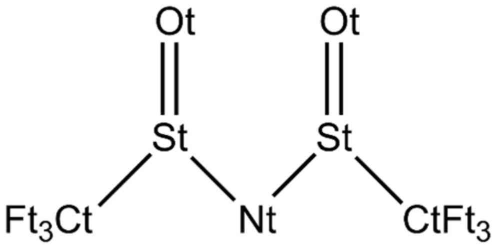

ANGLES	K_θ [kcal/mole/rad^2]	θ [deg]
Ft-Ct-Ft	93.3	107.1
St-Ct-Ft	82.9	111.8
Ct-St-Ot	104.0	102.6
Ot-St-Ot	115.8	118.5
Ot-St-Nt	94.3	113.6
Ct-St-Nt	97.5	100.2
St-Nt-St	80.2	125.6
Of-Sf-Of	95.5	119.3
Of-Sf-Nf	104.0	112.6
Sf-Nf-Sf	87.5	126.0
Ff-Sf-Nf	108.0	102.2
Ff-Sf-Of	128.5	103.9

FSI

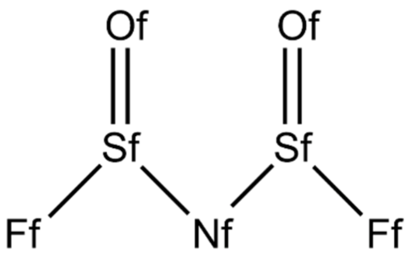

DIHEDRALS	$K_{\xi,n}$ [kcal/mole]	n	γ [deg]
Ft-Ct-St-Ot	0.1730	3	0.0
St-Nt-St-Ot	-0.0018	3	0.0
Ft-Ct-St-Nt	0.1580	3	0.0
St-Nt-St-Ct	3.9160	1	0.0
St-Nt-St-Ct	-1.2450	2	180.0
St-Nt-St-Ct	-0.3820	3	0.0
Of-Sf-Nf-Sf	-0.0020	3	0.0
Ff-Sf-Nf-Sf	0.3210	1	180.0
Ff-Sf-Nf-Sf	-1.7250	2	180.0
Ff-Sf-Nf-Sf	-0.3945	3	180.0
Ff-Sf-Nf-Sf	0.2480	4	180.0

VDW	ε [kcal/mole]	r_{min} [Å]
Nt	0.051	1.8240
St	0.075	2.2910
Ot	0.063	1.9440
Ct	0.020	1.7680
Ft	0.016	1.4900
Nf	0.145	1.8728
Of	0.090	1.6804
Ff	0.037	1.8071
Sf	0.150	2.3283

ELECTRO-STATIC	q [e]	α [Å^3]
Nt	-0.4854	1.45
St	0.5928	0.50
Ot	-0.3893	1.36
Ct	0.1391	1.05
Ft	-0.0702	0.60
Nf	-0.5218	1.45
Of	-0.3950	1.36
Ff	-0.1911	0.60
Sf	0.7420	0.50

Drude particles were not used for hydrogen atoms.

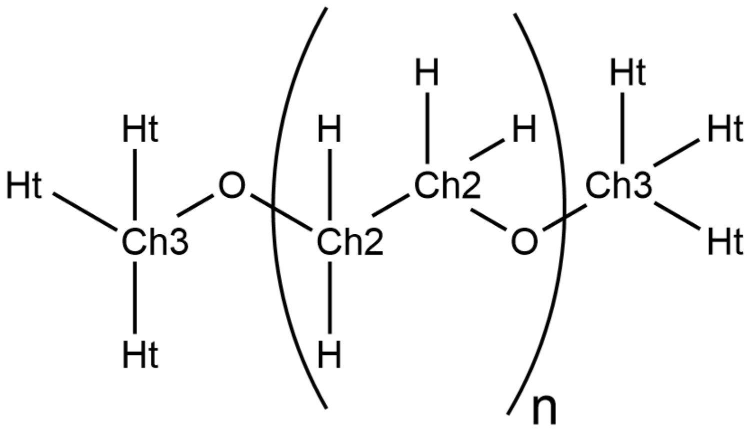

MONOGLYME (n = 1)
TETRAGLYME (n = 4)
PEO (n = 99)

MONOGLYME

BONDS	K_r [kcal/mole/Å^2]	r_0 [Å]
Ch2-Ch2	268.0	1.529
Ch2-O	320.0	1.410
Ch2-H	340.0	1.090
O-Ch3	320.0	1.410
Ht-Ch3	340.0	1.090

ANGLES	K_θ [kcal/mole/rad^2]	θ [deg]
Ch2-Ch2-O	50.0	109.5
Ch2-Ch2-H	37.5	110.7
Ch2-O-Ch2	60.0	109.5
Ch2-O-Ch3	60.0	109.5
O-Ch2-H	35.0	109.5
H-Ch2-H	33.0	107.8
O-Ch3-Ht	35.0	109.5
Ht-Ch3-Ht	33.0	107.8

DIHEDRALS	$K_{\xi,n}$ [kcal/mole]	n	γ [deg]
Ch2-Ch2-O-Ch2	0.383	3	0.0
Ch2-Ch2-O-Ch2	0.100	2	180.0
Ch2-Ch2-O-Ch3	0.383	3	0.0
Ch2-Ch2-O-Ch3	0.100	2	180.0
H-Ch2-O-Ch2	0.383	3	0.0
O-Ch2-Ch2-O	0.144	3	0.0
O-Ch2-Ch2-O	1.175	2	0.0
O-Ch2-Ch2-H	0.234	3	0.0
H-Ch2-Ch2-H	0.159	3	0.0
Ch2-O-Ch3-Ht	0.383	3	0.0
H-Ch2-O-Ch3	0.380	3	0.0

VDW	ε [kcal/mole]	r_{min} [Å]
Ch3	0.066	1.9640
Ch2	0.066	1.9640
O	0.140	1.6280
Ht	0.030	1.4030
H	0.030	1.4030

ELECTRO-STATIC	q [e]	α [$Å^3$]
Ch3	0.11	2.21
Ch2	0.14	1.76
O	-0.40	1.13
Ht	0.03	0.00
H	0.03	0.00

Drude particles were not used for hydrogen atoms.

TETRAGLYME/PEO

BONDS	K_r [kcal/mole/$Å^2$]	r_0 [Å]
Ch2-Ch2	268.0	1.529
Ch2-O	320.0	1.410
Ch2-H	340.0	1.090
O-Ch3	320.0	1.410
Ht-Ch3	340.0	1.090

ANGLES	K_θ [kcal/mole/rad^2]	θ [deg]
Ch2-Ch2-O	50.0	109.5
Ch2-Ch2-H	37.5	110.7
Ch2-O-Ch2	60.0	109.5
Ch2-O-Ch3	60.0	109.5
O-Ch2-H	35.0	109.5
H-Ch2-H	33.0	107.8
O-Ch3-Ht	35.0	109.5
Ht-Ch3-Ht	33.0	107.8

DIHEDRALS	$K_{\xi,n}$ [kcal/mole]	n	γ [deg]
Ch2-Ch2-O-Ch2	0.383	3	0.0
Ch2-Ch2-O-Ch2	0.100	2	180.0
Ch2-Ch2-O-Ch3	0.383	3	0.0
Ch2-Ch2-O-Ch3	0.100	2	180.0
H-Ch2-O-Ch2	0.383	3	0.0
O-Ch2-Ch2-O	0.144	3	0.0
O-Ch2-Ch2-O	1.000	2	0.0
O-Ch2-Ch2-H	0.156	3	0.0
H-Ch2-Ch2-H	0.156	3	0.0
Ch2-O-Ch3-Ht	0.383	3	0.0
H-Ch2-O-Ch3	0.383	3	0.0

VDW	ε [kcal/mole]	r_{min} [Å]
Ch3	0.066	1.9640
Ch2	0.066	1.9640
O	0.140	1.6280
Ht	0.030	1.4030
H	0.030	1.4030

ELECTRO-STATIC	q [e]	α [Å^3]
Ch3	0.11	2.21
Ch2	0.14	1.76
O	-0.40	1.13
Ht	0.03	0.00
H	0.03	0.00

Drude particles were not used for hydrogen atoms.

Na^+

VDW	ε [kcal/mole]	r_{min} [Å]
Na	0.0005	2.2842
Na-Ot	0.0056	3.9500
